# Extracellular vesicles in bone aging: therapeutic strategies and applications

**DOI:** 10.20517/evcna.2025.58

**Published:** 2025-11-10

**Authors:** Lin Tang, Peng Wang, Shihao Sheng, Huijian Yang, Yingying Jiang, Yingying Jing, Han Liu, Jiacan Su

**Affiliations:** ^1^Institute of Translational Medicine, Shanghai University, Shanghai 200444, China.; ^2^MedEng-X Institutes, Shanghai University, Shanghai 200444, China.; ^3^Organoid Research Center, Shanghai University, Shanghai 200444, China.; ^4^Department of Orthopedics, Xinhua Hospital, Shanghai Jiao Tong University School of Medicine, Shanghai 200092, China.; ^5^Department of Rehabilitation Medicine, Shanghai Zhongye Hospital, Shanghai 200941, China.; ^6^The Second Hospital of Sanming, Sanming 366013, Fujian, China.; ^7^Suzhou-Shanghai University Innovation Center, Shanghai University, Suzhou 215000, Jiangsu, China.; ^8^National Center for Translational Medicine (Shanghai) SHU Branch, Shanghai University, Shanghai 200444, China.; ^#^These authors contributed equally to this work.

**Keywords:** Extracellular vesicle, bone aging, osteoporosis, osteoarthritis, engineering modification

## Abstract

As global aging intensifies, the issue of bone aging has become increasingly prominent. Osteoporosis and osteoarthritis, which are common complications of bone aging, significantly impair patients’ quality of life due to their high prevalence and disability rates, thereby presenting a major public health challenge. Extracellular vesicles (EVs), nanoscale particles released by cells, are regarded as an ideal platform for bone aging due to their high biocompatibility, ease of modification, and significant therapeutic efficacy. This review provides a comprehensive summary of the latest advancements in mammalian-, bacterial-, and plant-derived EVs, particularly in the context of bone aging. Furthermore, organoids, as lab-grown models replicating organ physiology, produce organoid-derived EVs that represent an especially promising avenue for therapeutic application. This review focuses on exploring potential therapeutic strategies that capitalize on the unique advantages of each EV type for treating bone aging. It is anticipated that a thorough comprehension of these EV types will unveil new avenues for bone aging treatment.

## INTRODUCTION

As individuals age, bone aging becomes a natural physiological process, characterized by the deterioration of bone function and a reduction in bone density^[1]^. These changes are a consequence of bone metabolism, and an imbalance in this process can readily lead to the onset of bone diseases, particularly osteoporosis (OP) and osteoarthritis (OA)^[2]^. A survey by the World Health Organization projects that the global population aged 60 and above will increase from 900 million in 2015 to 2 billion by 2050^[3]^. Therefore, bone aging not only affects the physical health of the elderly but also significantly affects their quality of life, exerting substantial pressure on global healthcare systems^[4]^. Various therapeutic drugs have been developed to regulate bone homeostasis, including molecular-targeted drugs, selective estrogen receptor modulators, synthetic parathyroid hormone, calcitonin, and bisphosphonates^[5,6]^. Although drugs such as bisphosphonates and denosumab are widely used and effective in managing bone loss, they may carry risks such as atypical fractures, osteonecrosis of the jaw, and rebound bone loss upon discontinuation, which limit their long-term application in certain patient populations. Consequently, the development of novel therapeutic strategies to address bone aging-related diseases has become an urgent priority.

According to the International Society for Extracellular Vesicles, extracellular vesicles (EVs) are defined as membrane-bound particles that bud from cells spontaneously and lack the ability to replicate^[7]^. Earlier investigations have revealed that these vesicles encapsulate multiple biologically active components, including RNA and proteins. They are crucial in regulating cellular properties and play key roles in angiogenesis, immunomodulation, cell proliferation, and intercellular communication^[8,9]^. EVs have garnered significant attention among researchers, particularly those dedicated to the exploration of autoimmune diseases, cancer, and degenerative disorders^[10-13]^. Meanwhile, researchers have identified great potential for them to modulate bone metabolism^[5]^.

**Figure 1:**
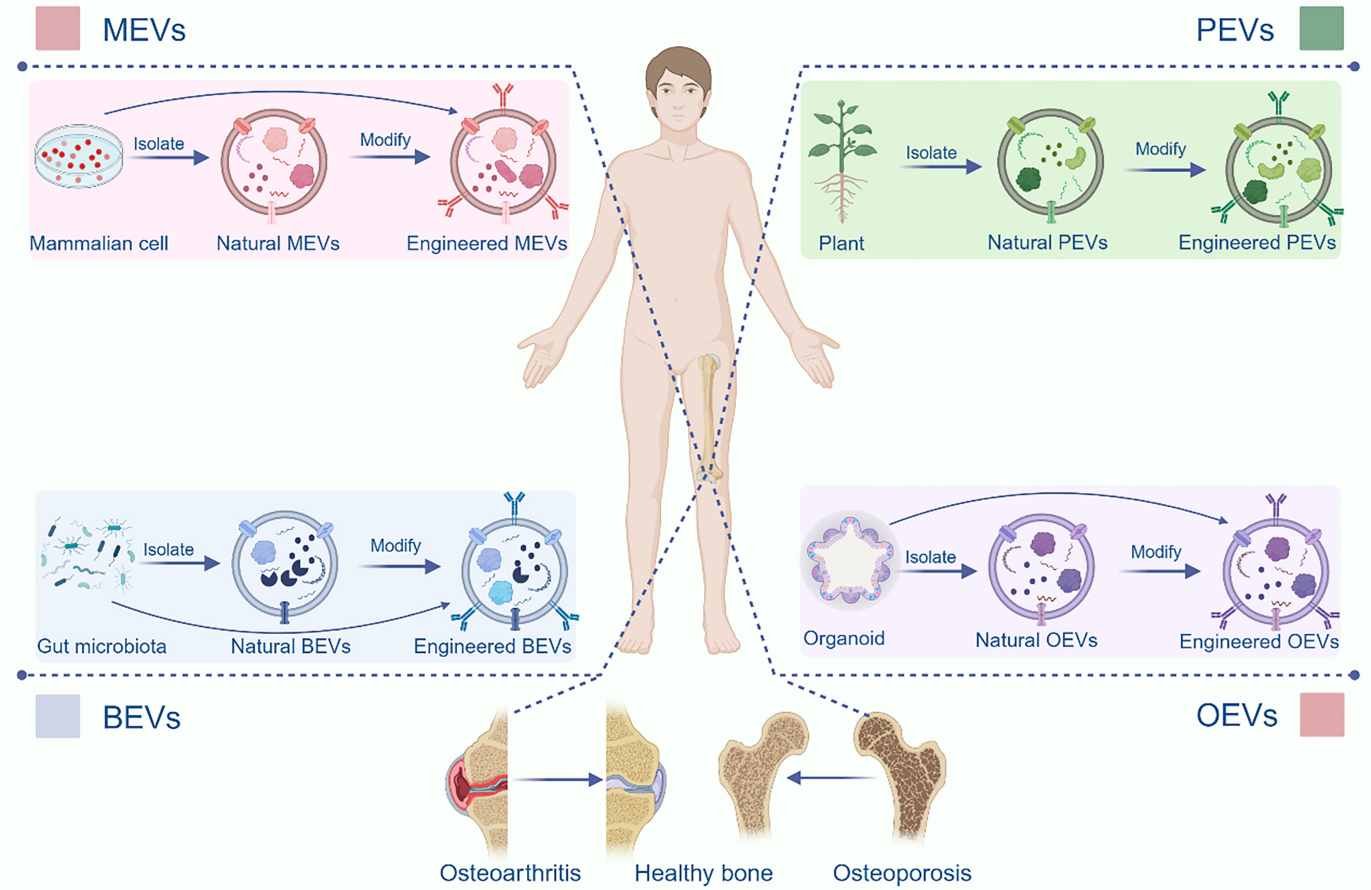
Both natural and engineered EVs offer significant promise in the treatment of bone aging. MEVs, BEVs, and PEVs can efficiently regulate bone metabolism, thus offering a promising therapeutic approach for related diseases. Based on existing research, we propose a novel therapeutic strategy utilizing OEVs. Created in BioRender. Bigbone B. (2025) https://BioRender.com/ldwwnlx.00. EVs: Extracellular vesicles; MEVs: mammalian extracellular vesicles; PEVs: plant-derived extracellular vesicles; BEVs: bacterial extracellular vesicles; OEVs: organoid-derived extracellular vesicles.

This review provides a comprehensive and systematic exploration of the generation, composition, structural characteristics, internalization mechanisms, and isolation techniques associated with the four distinct classes of EVs. Building on this foundational understanding, we delve into advanced engineering strategies for the functional modification of EVs, highlighting their potential to enhance therapeutic efficacy. Subsequently, we present an in-depth analysis of the latest research advancements in the application of EVs for the treatment of bone aging-related pathologies [Figure 1]. By elucidating the unique properties and functional roles of these four EV subtypes, this review aims to pave the way for the development of novel and targeted therapeutic interventions to address the multifaceted challenges of bone aging.

## OVERVIEW OF EVs

**Figure 2:**
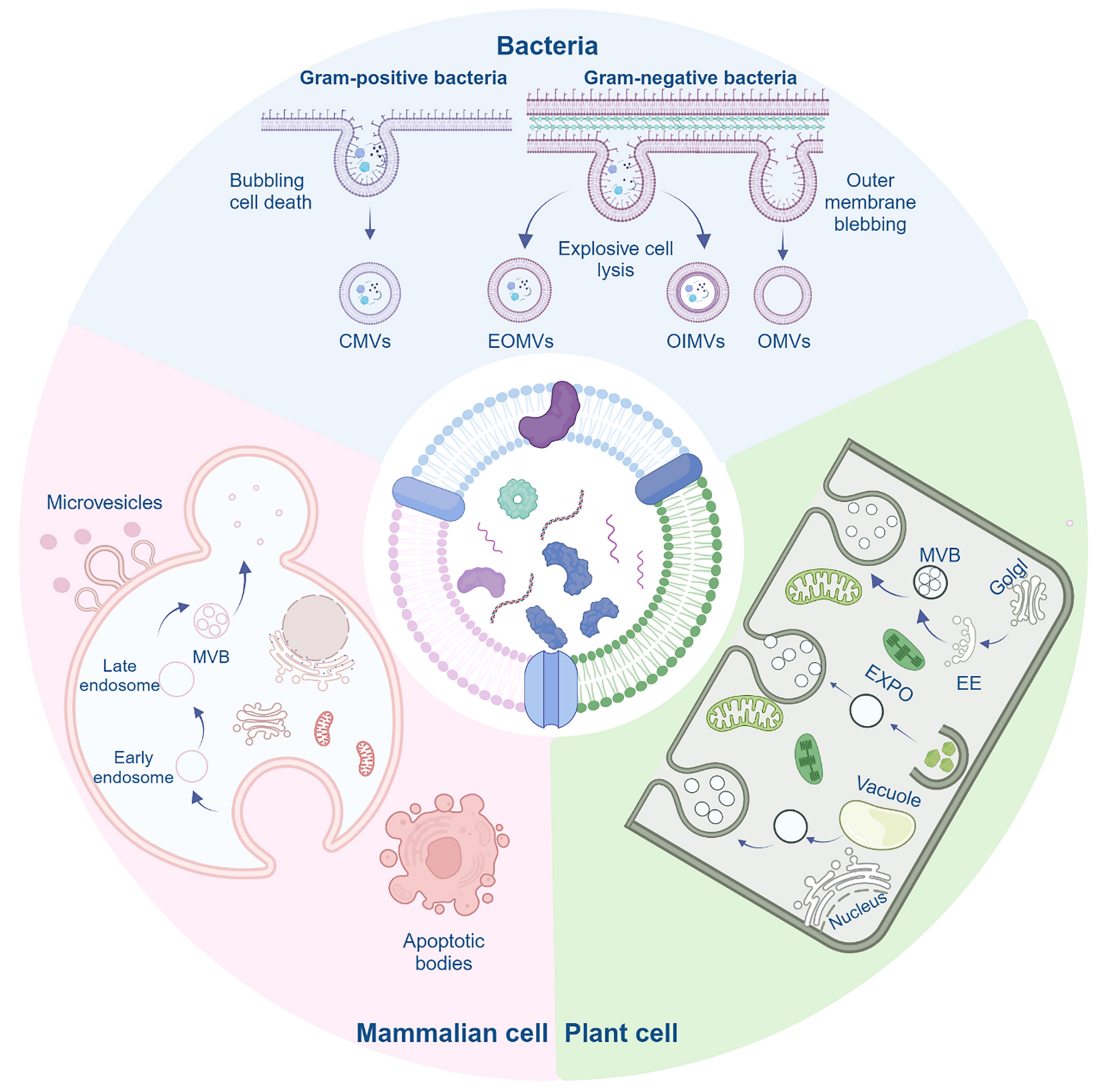
The biogenesis of BEVs, MEVs, and PEVs varies among different organisms. Gram-positive and Gram-negative bacteria produce EVs through distinct mechanisms. In Gram-positive bacteria, EVs are generated through a process known as bubbling cell death, specifically referred to as CMVs. Gram-negative bacteria employ two main mechanisms to produce BEVs: outer membrane blebbing, which results in OMVs, and explosive cell lysis, which leads to the formation of both OIMVs and EOMVs. MEVs are primarily produced via microvesicles, apoptotic vesicles, and the MVBs pathway, while PEVs are mainly produced through the EXPO pathway, vesicular pathway, and MVBs pathway. Created in BioRender. Bigbone B. (2025) https://BioRender.com/gxc0cps. BEVs: Bacterial extracellular vesicles; MEVs: mammalian extracellular vesicles; PEVs: plant-derived extracellular vesicles; CMVs: cytoplasmic membrane vesicles; OMVs: outer membrane vesicles; OIMVs: outer-inner membrane vesicles; EOMVs: explosive outer membrane vesicles; MVBs: multivesicular bodies; EXPO: exocyst-positive organelle; EE: early endosome.

EVs serve as a designation for nanovesicles secreted by cells, characterized by a phospholipid bilayer structure^[14,15]^. In this section, we furnish an exhaustive overview detailing the mechanisms governing the generation, structure, composition, isolation, and internalization processes associated with diverse types of EVs. We will present their generation mechanisms separately [Figure 2].

### Generation mechanism of MEVs

Based on the process of formation and biological properties, we classified mammalian-derived EVs (MEVs) into three categories, namely microvesicles, apoptotic vesicles, and exosomes^[16]^. Microvesicles form directly through cell membrane outgrowth, whereas apoptotic vesicles originate from cell fragmentation during apoptosis. The formation of exosomes is relatively complex and has received great attention due to their unique biological functions^[17]^. Here, we use MEVs to represent exosomes. The formation of MEVs can be roughly divided into three stages. Initially, the plasma membrane undergoes invagination, giving rise to endocytosed vesicles, which then undergo fusion to generate early endosomes. Following this, endocytosed vesicles undergo invagination to encapsulate intracellular cargo, forming multiple intraluminal vesicles (ILVs). These ILVs are subsequently transformed into multivesicular bodies (MVBs), which then fuse with the plasma membrane, releasing their contents into the extracellular space^[18]^. In previous studies, researchers explored and conclusively identified the involvement of RAB proteins, cytoskeletal components (actin and microtubule proteins), synaptic binding proteins, and Soluble NSF Attachment Protein REceptor (SNARE) proteins in the secretion process of EVs^[19-21]^. Furthermore, recent investigations have revealed that phosphatidylinositol plays a regulatory role in the secretion of EVs^[22]^.

### Generation mechanism of BEVs

Bacterial-derived EVs (BEVs) were initially identified as a result of outer membrane blistering in Gram-negative bacteria^[23]^. For an extended period, there was a prevailing belief among researchers that these vesicles were restricted to Gram-negative bacteria. Nevertheless, notably, BEVs from Gram-positive bacteria, particularly *Staphylococcus aureus*, have been successfully isolated from culture supernatants^[24]^. Subsequently, BEVs have also been observed in *Streptomyces coelicolor* and *Bacillus subtilis* using transmission and scanning electron microscopy^[25,26]^.

#### Gram-positive bacteria

In previous studies, three hypotheses have been proposed regarding the generation of EVs by Gram-positive bacteria. The first is that the cell wall is partially degraded by proteases, resulting in cell wall laxity and a larger pore size for the release of EVs^[24,27]^. The second suggests that EVs utilize protein channels in the peptidoglycan layer to pass through the extracellular environment^[28,29]^. The third involves the generation of a specific pressure by EVs during their formation by the cell membrane. This pressure facilitates the traversal of EVs through the aperture of the cell wall. Concurrently, the size of the cell wall aperture exerts a measure of control over the dimensions of EVs^[28,29]^. Nevertheless, contemporary studies propose that Gram-positive bacteria are generated through bubbling cell death^[30]^. The general principle is that the peptidoglycan layer is weakened or degraded for various reasons, thereby allowing the cytoplasmic membrane vesicles (CMVs) produced by the cytoplasmic membrane to be readily released. This phenomenon has been exemplified, as seen with endolysin and β-lactam antibiotics, which weaken the peptidoglycan layer^[30,31]^. We postulate that other enzymes capable of disrupting the cell wall may similarly induce bubbling cell death, thereby contributing to the production of EVs.

#### Gram-negative bacteria

Contemporary research indicates that Gram-negative bacteria utilize two primary production mechanisms: outer membrane blebbing and explosive cell lysis^[30]^. The introduction of hydrophobic molecules or involvement in peptidoglycan biosynthesis within the outer membrane induces destabilization, resulting in the formation of classical outer membrane vesicles (OMVs)^[23,30]^. The production of BEVs is profoundly influenced by genetic background and growth conditions. The literature indicates that administration of either gentamicin or polymyxin disrupts lipid homeostasis, thereby inducing extracellular membrane stress and promoting OMV formation through membrane blebbing^[32,33]^. In addition, the occurrence of explosive cell lysis is associated with the attenuation of endolysin activity, causing degradation of the peptidoglycan layer. Consequently, this weakening causes the inner membrane to protrude into the periplasm, triggering vesicle production. Within this mechanism, both outer-inner membrane vesicles (OIMVs) and explosive OMVs (EOMVs) may be generated^[30]^. Under the same production mechanism, OIMVs possess two membranes while EOMVs have only one. A growing body of evidence suggests that antibiotics can trigger the SOS response in cells, initiating the expression of endolysin and provoking explosive cell lysis^[34,35]^.

### Generation mechanism of PEVs

Compared with other EVs, the generation mechanism of plant-derived EVs (PEVs) has received limited attention in the research domain. A more comprehensive understanding of this mechanism necessitates further exploration by researchers. Three primary mechanisms have been proposed: the vesicle pathway, the MVB pathway, and the exocyst-positive organelle (EXPO) pathway^[36]^. It has been reported that organelles in southern mustard cells fuse with the plasma membrane, resulting in the production of single-membrane vesicles^[37]^. Additionally, vesicles released via the vacuole pathway usually contain defensive substances^[38]^. The MVB pathway is the primary route for PEV production, and it has been reported that MVBs can fuse with the plasma membrane to release vesicles in carrot cells^[39]^. Many subsequent studies have also shown that this phenomenon occurs in various plant cells, confirming the MVB pathway as the main mode of PEV production^[40]^.

### Structure and composition of EVs

#### MEVs

Microvesicles, apoptotic vesicles, and exosomes measure approximately 200-1,000 nm, 500-2,000 nm, and 40-160 nm in diameter, respectively^[41]^. MEVs primarily contain a diverse array of RNA and nucleic acids, with membranes rich in sphingolipids, cholesterol, and ceramides^[42,43]^. The intricacy of cargo sorting is noteworthy. Current findings suggest the involvement of crucial proteins, including CD81, CD63, tumor susceptibility gene 101 protein (TSG101), apoptosis-linked gene 2-interacting protein X (Alix), and the endosomal sorting complexes required for transport (ESCRT)^[41]^. TSG101, Alix, CD9, CD81, CD63, SNARE, and RAB GTPases are commonly used as markers to characterize successful EV extraction and to indicate the presence of EVs^[44]^.

#### BEVs

Structurally, Gram-positive bacteria have an inner layer consisting of a single lipid membrane and an outer layer containing peptidoglycan and phosphatidic acid^[45]^. The robust outer cell wall likely hinders the release of EVs. The first *Staphylococcus aureus* EVs were detected with diameters ranging from 20 to 100 nm^[24]^. Due to the diversity of bacterial species and distinct production mechanisms, we observed significant variation in the diameters of EVs produced by different bacteria^[25,46-49]^. EVs produced by Gram-positive bacteria contain various cargoes, such as endolysins, cytoplasmic membrane proteins, DNA, RNA, and virulence factors^[50]^. These distinct molecules may be responsible for eliciting varied biological functions. It is of significance that EVs from Gram-positive bacteria exhibit an absence of lipopolysaccharides. This distinction can be attributed to the inherent characteristic of lipopolysaccharides being a constituent of the cell wall in Gram-negative bacteria.

The structural composition of Gram-negative bacteria includes membrane structures such as an outer membrane, a cytoplasmic membrane, and a periplasmic space^[51]^. The structural makeup of the outer membrane consists of an outer leaflet made of lipopolysaccharides and an inner leaflet composed of phospholipids. In contrast, the cytoplasmic membrane is typically characterized by phospholipid bilayers. Concurrently, the periplasmic space encompasses layers of peptidoglycan, lipoproteins, and periplasmic proteins^[52]^. The reticular peptidoglycan layer within the periplasm bestows bacteria with their distinctive morphology and serves as a protective shield against absolute pressure and osmotic variations. Evidently, Gram-negative bacteria have undergone more thorough investigation in comparison to Gram-positive counterparts. The EVs produced under different mechanisms are not quite the same due to different production mechanisms. Abundant studies have substantiated the existence of DNA, RNA, periplasmic proteins, and cytoplasm encapsulated within OMVs^[53,54]^. In contrast, both OIMVs and EOMVs demonstrate a somewhat greater cargo capacity, including a diverse array of cytoplasmic components such as DNA, RNA, phages, hydrophobic molecules, cytoplasmic membrane proteins, outer membrane proteins, endolysins, and virulence factors^[55,56]^.

#### PEVs

PEVs are bilayer vesicles ranging from 30 to 500 nm and contain a variety of nucleic acids, proteins, and metabolites^[57]^. Although related studies are limited, it is known that water channel proteins, membrane junctional proteins, and heat shock proteins play a role in the biogenesis of PEVs^[58]^. PEVs are rich in lipids such as phosphorylcholine, phosphatidic acid, and phosphatidylethanolamine, which are important for various biological functions and intercellular interactions^[59]^. Additionally, PEVs sourced from distinct sources exhibit a diverse array of metabolites. For instance, those derived from fruits may encompass vitamin C^[60]^. Ginger-derived PEVs have been reported to encapsulate bioactive constituents, including 6-gingerol and 10-gingerol, which exert anti-inflammatory and anti-cancer effects through the modulation of the nuclear factor-kappa B (NF-κB) and phosphatidylinositol 3’-kinase/Akt (PI3K/Akt) signaling pathways^[61]^. Furthermore, accumulating evidence indicates that PEVs can transport plant-derived microRNAs (miRNAs), such as miR-168, into mammalian cells, thereby regulating gene expression associated with inflammation and tumor progression^[62]^.

### Isolation of EVs

To better advance related research, a fast and effective method for extracting EVs is essential. MEVs are widely present in body fluids; BEVs can be extracted from culture medium, and PEVs are often obtained from plant sap. However, implementing these methods in practice presents significant challenges.

#### MEVs

**Figure 3:**
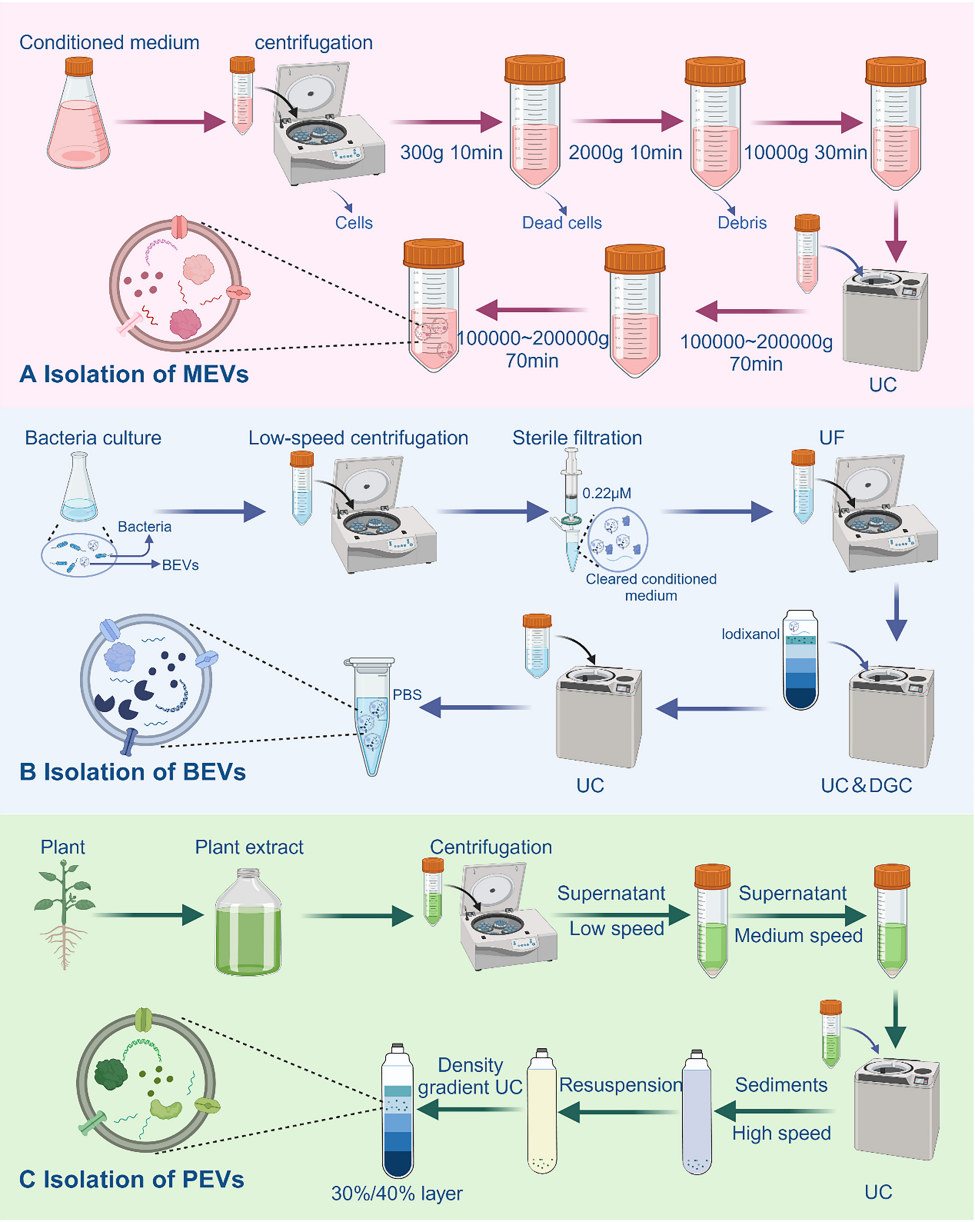
Overview of EV Isolation. (A) Isolation of MEVs. The process involves ultracentrifugation of pretreated samples, preceded by low-speed centrifugation to remove impurities such as cells and cellular debris; (B) Isolation of PEVs. After pretreatment, plant juice undergoes a sequential centrifugation process at low, medium, and high speeds. PEVs are then obtained from the liquid layer using the discontinuous sucrose gradient method, as shown in the figure; (C) Isolation of BEVs. Samples are first centrifuged at low speed and filtered to remove bacteria. The filtered supernatant is then concentrated using ultrafiltration membranes. BEVs are isolated by two rounds of ultracentrifugation and finally resuspended in PBS. Created in BioRender. Bigbone B. (2025) https://BioRender.com/zavrv7g. EVs: Extracellular vesicles; MEVs: mammalian extracellular vesicles; BEVs: bacterial extracellular vesicles; PEVs: plant-derived extracellular vesicles; UC: ultracentrifugation; DGC: density gradient centrifugation; UF: ultrafiltration; PBS: phosphate-buffered saline.

We summarize the current methods for isolating EVs, including precipitation, density gradient centrifugation (DGC), affinity separation, ultracentrifugation (UC), ultrafiltration (UF), and size exclusion chromatography (SEC). Various separation methods present distinct advantages and drawbacks. According to the first global survey of commonly used techniques, more than 80% of researchers favored UC, including differential centrifugation, as their primary method for isolating MEVs^[63]^. In a recent study, Wu *et al*. achieved temporal separation of EVs by introducing azide compounds as timestamps for click chemistry labeling in combination with microfluidics^[64]^. Li *et al*. summarize the most commonly used isolation methods^[65]^. The isolation of MEVs primarily relies on the combined use of differential centrifugation and UC. [Figure 3A]. Low-speed centrifugation is commonly used to remove dead cells, cellular debris, and other impurities, whereas UC is conventionally employed for the specific isolation of MEVs. Iodixanol is often applied to obtain more purified MEVs.

#### BEVs

Choosing different separation methods based on the characteristics of bacteria and culture media can enable strategic integration, leading to optimal yield and concentration. For example, in the cultivation process aimed at obtaining Akkermansia muciniphila (AKK), the fermentation medium for this strain is enriched with porcine mucin. Nevertheless, the surplus mucin presents challenges, notably heightened protein viscosity, thus introducing complexities into the isolation and extraction procedures of BEVs^[66]^. Liu *et al*. successfully isolated AKK-derived BEVs using a combination of UC, UF, and DGC techniques^[67]^. Furthermore, the growth requirements of specific bacteria, such as *Helicobacter pylori*, demand more stringent conditions. Although we were able to isolate the BEVs using the above methods, the microvesicles contained therein were difficult to isolate completely^[41]^. To address the complex culture conditions required by certain bacteria, we have developed a protocol suitable for a wide range of bacterial species. By applying this isolation protocol, BEVs were successfully isolated from strains such as *E. coli Nissle 1917* and AKK [Figure 3B].

#### PEVs

Standard separation methods for PEVs are still not established, and to obtain high-quality PEVs, most current protocols are based on those for MEVs. At present, the combination of multiple separation methods to separate PEVs remains the best solution [Figure 3C]. Zhan *et al*. obtained PEVs of Pueraria lobata origin using a combination of UC and UF^[68]^. Interestingly, Hwang *et al*. also successfully isolated PEVs of yam origin using UC at different speeds^[69]^. It is worth mentioning that Yang *et al*. developed a method that combines electrophoresis with dialysis and successfully isolated PEVs from lemon juice^[70]^. In this strategy, separation is achieved by driving RNAs and proteins toward the cathode and anode under an electric field, while PEVs were confined in a dialysis bag. Most importantly, the number of PEVs isolated by this method is comparable to that obtained by UC and does not require expensive equipment. Without a doubt, the amalgamation of electrophoresis and dialysis emerges as an exceedingly promising method for the separation of PEVs.

### Internalization of EVs

**Figure 4:**
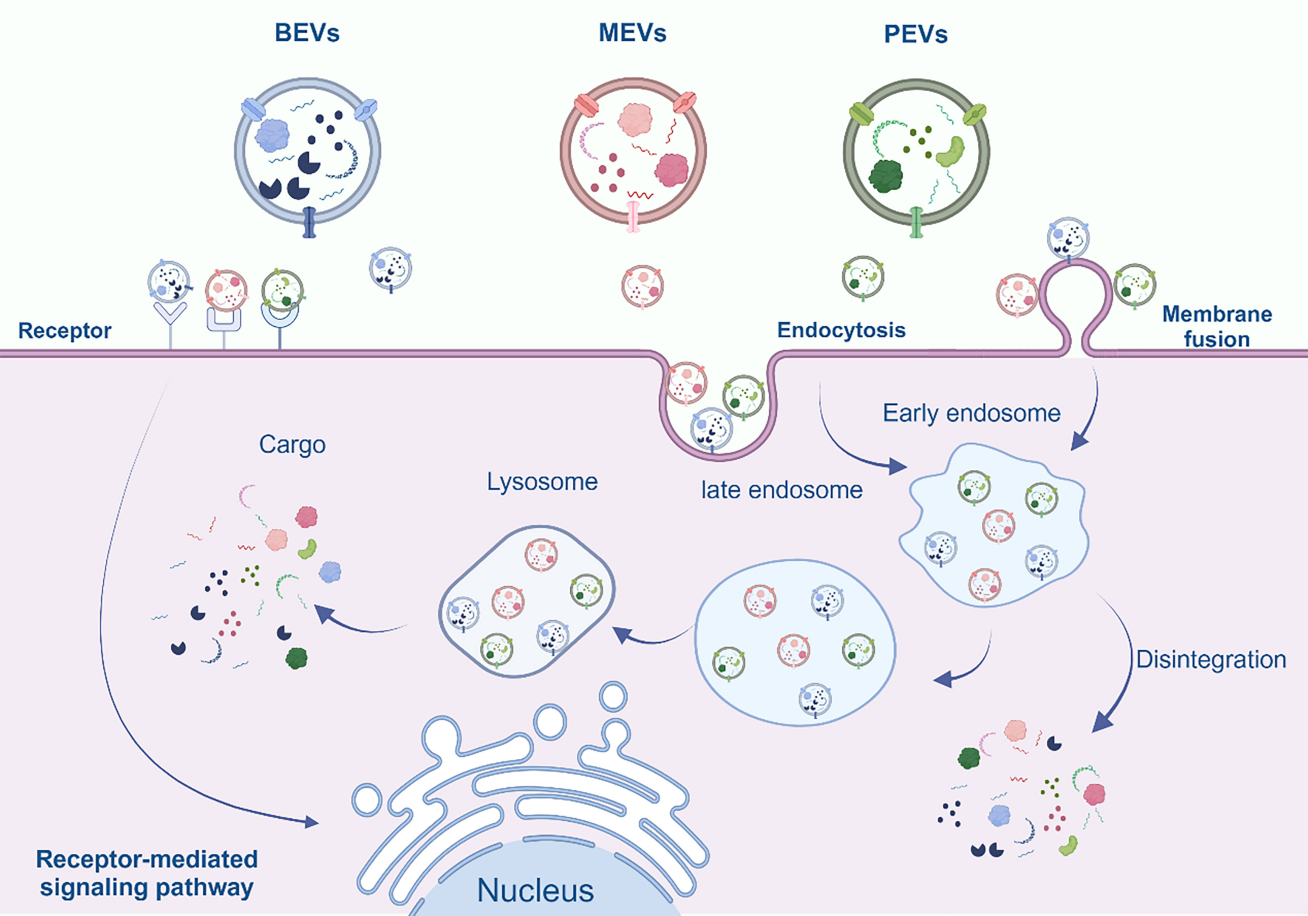
Overview of internalization of MEVs, BEVs and PEVs. Cells uptake them through three principal mechanisms: membrane fusion, endocytosis, and receptor-mediated signaling. Subsequently, once inside the cell, they release their contents, fulfilling their designated biological functions. Created in BioRender. Bigbone B. (2025) https://BioRender.com/e93q0ii. EVs: Extracellular vesicles; MEVs: mammalian extracellular vesicles; BEVs: bacterial extracellular vesicles; PEVs: plant-derived extracellular vesicles.

A wealth of compelling evidence suggests that internalization of MEVs, BEVs, and PEVs generally does not induce cytotoxicity^[58,60,71,72]^. The composition and structural features of diverse EVs demonstrate resemblances, so it is reasonable to speculate that the internalization process of EVs is roughly the same. Based on previous findings, we conclude that there are three main modes of internalization of EVs, including endocytosis, membrane fusion, and receptor-mediated signaling [Figure 4]^[73]^. Recent evidence indicates that phagocytosis constitutes an additional route of EV internalization, occurring predominantly in phagocytic cells, including macrophages, neutrophils, and dendritic cells (DCs)^[74]^. The pathway by which EVs are internalized depends on both their cargo and surface structure. Within this array of mechanisms, receptor-mediated signaling stands out as a primary mode of cellular communication. It involves interactions with cells harboring specific receptors, facilitating the transmission of information, elicitation of responses, and regulation of signaling pathways. The pivotal receptors in this context are predominantly toll-like receptors (TLRs)^[75]^. The membrane fusion arises from the fundamental characteristic of EVs as phospholipid bilayer structures. This distinctive attribute empowers EVs to undergo direct membrane fusion with cells sharing a comparable structural composition, thereby facilitating their seamless entry into the target cells. Finally, the intricate process of endocytosis is broadly categorized into specific and non-specific types. Diverse modalities within this mechanism engage various biomolecules. In specific endocytosis, lattice proteins, vesicle proteins, dynamin, and cholesterol play a facilitating role^[76,77]^. In this approach, actin-based cellular protrusions, called filopodia, act as "endocytosis hotspots" for EV uptake. EVs move along retractile fibers and filopodia toward the target cell before being endocytosed^[78]^. In this manner, endocytosis can span a period of up to 20 min^[79]^. Non-specific endocytosis is broadly categorized into cytosolic drinking, heparan sulphate proteoglycan (HSPG)-mediated endocytosis, and lipid raft-mediated endocytosis^[80]^. The primary form of endocytosis entails the degradation of foreign cargo through fusion with lysosomes. Therefore, this approach is acknowledged as a mechanism employed by immune cells to break down non-specific antigens^[81]^. The latter two pathways, although classified as non-specific uptake mechanisms, reveal a certain degree of selectivity influenced by the varying abundance of HSPG and lipid rafts on specific cells in comparison to others^[82,83]^.

## ENGINEERING OF EVs

**Figure 5:**
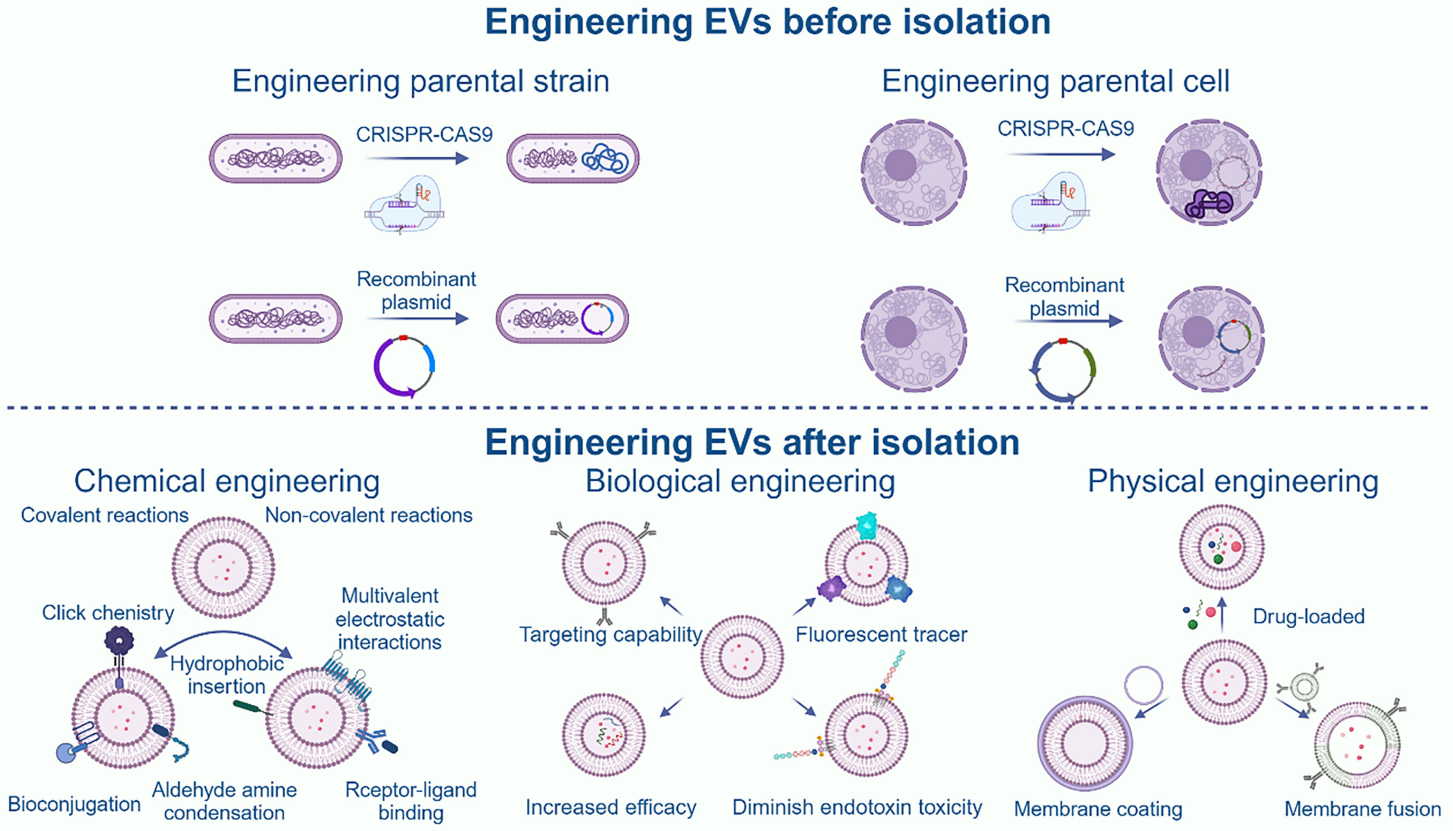
Overview of engineering modifications to EVs. It is divided into modification of parental strains, physical modification, chemical modification, and biological modification. Created in BioRender. Bigbone B. (2025) https://BioRender.com/sy9pesy. EVs: Extracellular vesicles; CRISPR-Cas9: clustered regularly interspaced short palindromic repeats-associated protein 9.

EVs have potential applications in bone disease therapy; however, their mass production is challenging due to various limitations, such as the low yield of MEVs. To address this, we focused on BEVs. To obtain better immunogenicity, we focused on PEVs. However, EVs still face some common limitations, the most obvious being a lack of targeting ability, low therapeutic efficiency, and poor pharmacokinetics. These challenges can be addressed through engineering modifications [Figure 5]. In addition, we propose using artificial intelligence (AI) to assist in EV modification.

### Engineering parental strain

Due to the distinctive biogenesis mechanisms associated with BEVs, including explosive cell lysis and bubbling cell death, there is the potential for BEVs to access the contents within the parent bacteria. In recent years, Tang *et al*. introduced the concept of materials synthetic biology, a conceptual framework that amalgamates the engineering principles of materials science and synthetic biology. This integration systematically transforms living systems into dynamic and responsive materials with emergent and programmable functions^[84]^. Combined with the unique advantages of BEVs, this integration makes BEVs full of possibilities in enhancing therapeutic OA capabilities. Chen *et al*., Li *et al*. and Cheng *et al*. have applied synthetic biology strategies to apply BEVs in the antitumor field and have achieved success^[71,85,86]^. In the engineering of parental strains, two main strategies are overexpression and knockout^[72]^. Among the overexpression strategies, the focus is primarily on either bone therapeutic effectors or bone-targeting effectors.

Currently, specific effectors can be overexpressed or knocked down based on clustered regularly interspaced short palindromic repeat (CRISPR)-Cas9 and plasmid-based modification techniques. CRISPR/Cas9 genome editing enables producer cells to stably express bone-targeting ligands (SDSSD, E7, or bisphosphonate peptides) or therapeutic proteins on EV membranes via lysosome-associated membrane protein 2b (LAMP2B) or CD63 fusion constructs^[87]^. EVs can also be engineered to deliver transient CRISPR complexes for *in vivo* editing, with rigorous off-target evaluation imperative^[88]^. Although bone morphogenetic protein-2 (BMP-2) is a potent osteoinductive agent, its clinical use has been linked to ectopic ossification, inflammatory responses, and osteolytic reactions. To mitigate these risks in EV-based delivery platforms, potential strategies include localized controlled release systems (EV-loaded hydrogels), bone-homing ligand modification to reduce systemic exposure, membrane-anchored BMP-2 display to restrict diffusion, using inducible or transient expression systems in producer cells, and substituting BMP-2 with pro-osteogenic miRNAs or small molecules to reduce ectopic ossification risk. Vascular endothelial growth factor (VEGF) and BMP-2 are typical bone therapeutic effectors. BMP-2 significantly promotes osteoblast differentiation in bone marrow mesenchymal stem cells (BMSCs)^[89]^. Hay *et al*. orchestrated the growth of stem cells and the process of osteogenesis by strategically overexpressing BMP-2 within engineered *Lactococcus lactis*^[90]^. Likewise, VEGF serves as a potent factor in vascular growth, making a substantial contribution to the differentiation of osteoblasts^[91]^. Lu *et al*. also applied *Lactococcus lactis* overexpressing VEGF to regulate angiogenesis^[92]^. The CXC chemokine receptor 4 (CXCR4) possesses bone-targeting capabilities, with its ligand being stromal cell-derived factor 1 (SDF1) expressed by BMSCs^[93]^. Cheng *et al*. devised a display system utilizing Cly A, enabling the presentation of multiple proteins on the membrane surface of BEVs^[86]^. Thus, receptors or ligands similar to CXCR4 and SDF1 will be displayed by the Cly A display system, potentially providing access to bone-targeting-competent BEVs. The CRISPR-Cas9 and λ-red recombination systems are commonly used tools in the knockout strategy. It has been confirmed that deleting specific genes (*msbA, msbB, lpxM, and lpxL1*) also results in the production of low-toxicity BEVs^[72]^. Thomas *et al*. formulated a strain wherein the *nlpL* gene was deliberately removed, significantly amplifying the production of BEVs^[94]^.

Analogous strategies have been applied to parental plant strains, where editing tools are used to eliminate or introduce plant-specific effectors^[95]^. Researchers anticipate that transgenic plants could produce PEVs with tailored functions. However, challenges such as low editing rates, protracted growth periods, off-target effects, and the robustness of plant cell walls have temporarily hindered the realization of this groundbreaking concept^[96]^.

### Biological engineering

At present, the genetic engineering of EVs can be comprehensively classified into three primary methodologies. First, the application of fluorescent or radioactive tracer technology facilitates accurate *in vivo* tracking of EVs^[97]^. Subsequent to this, the integration of gene sequences encoding therapeutic factors or specific lipopolysaccharides is utilized to adjust and augment the therapeutic effects of EVs^[98,99]^. Lastly, engineering is carried out through the incorporation of specific targeting ligands, thereby exhibiting the corresponding ligands on the membrane surface and endowing EVs with targeted functionality^[100]^. Genetic engineering of EVs is primarily achieved through methodologies including electroporation transfection, lentiviral transfection, and liposome transfection^[98,100,101]^.

Lai *et al*. used a combination of Gaussia luciferase and metabolic biotinylation to create an EV for multimodal imaging, which was able to demonstrate the distribution and metabolism of EVs *in vivo*, providing a new approach for subsequent pharmacological studies^[97]^. Zhang *et al*. obtained lentiviral-transfected EVs with high Syndecan-1 expression, and EVs played a good therapeutic role in a lipopolysaccharide-stimulated acute lung injury model^[102]^. The overexpression strategy is also very practical in BEVs, as mentioned above. Endowing EVs with targeting ability has been a hot topic of research, and LAMP2B is the most commonly used related protein. Due to the adaptability of the N-terminus of LAMP2B in accepting diverse targeting sequences, researchers have documented a range of outcomes. Notably, fusing human epidermal growth factor receptor 2 with the N-terminus of LAMP2B demonstrated targeted binding for colorectal tumor applications^[103]^. The cardiac-targeting peptide, upon binding to the N-terminus of LAMP2B, intensifies the targeting of cardiac tissue. Simultaneously, the attachment of cartilage affinity peptides to the N-terminus of LAMP2B enhances its ability for cartilage targeting^[100,104]^. Notably, the transmembrane protein CD63 has also been a hotspot for researchers. Liang *et al*. engineered MEVs with hepatocellular carcinoma-targeting ability by expressing a gene fusion between the transmembrane protein CD63 and the Apo-A1 sequence in 293T cells^[105]^.

### Chemical engineering

In the realm of chemical engineering, both covalent and non-covalent reactions are widely employed methodologies. Non-covalent interactions primarily include receptor-ligand binding, multivalent electrostatic interactions, and hydrophobic insertion. Meanwhile, covalent reaction methods provide key strategies, including bioconjugation, aldehyde-amine reactions, and click chemistry.

Receptor-ligand interactions rely on the specific recognition between receptors and their corresponding ligands, enabling targeted binding and signaling. Liu *et al*. designed a technique using dip-pen nanolithography to generate lipid membrane microarrays loaded with specific antibodies, including biotin antibodies and streptavidin^[106]^. This approach enables the selective and efficient separation of EVs. Yang *et al*. devised a pH-responsive superparamagnetic nanoparticle cluster, capitalizing on the specific recognition between transferrin and the transferrin receptor. This specificity facilitated the successful isolation of transferrin receptor-positive EVs from blood^[107]^. The mechanism of multivalent electrostatic interactions hinges on the interplay between positive and negative electrostatic attractions^[108]^. Due to the inherently negatively charged nature of EV membranes, Tamura *et al*. used a simple mixture of cationic branched-chain starch with EVs. Their investigations unveiled that these EVs exhibited an expedited internalization into HepG2 cells and an enhanced affinity for the liver^[108]^. Nakase *et al*. increased cytoplasmic delivery of EVs through co-treatment with a pH-sensitive fusion peptide and cationic lipids^[109]^. Hydrophobic insertion is commonly employed due to the predominant phospholipid bilayer composition in the membrane structure of EVs. Certain biomaterials or functional groups, with hydrophilic heads and hydrophobic tails, can be directly inserted into the EV bilayer through co-incubation^[110]^. The compound 1, 2-Distearoyl-sn-glycero-3-phosphoethanolamine-Poly(ethylene glycol) (DSPE-PEG) is widely acknowledged as the commonly utilized amphiphilic molecule across diverse applications. It can be linked with diverse functional ligands, such as folic acid, biotin, and arginyl-glycyl-aspartic acid, thereby enhancing the targeting ability of EVs.

Bioconjugation is generally based on the covalent binding of two molecules. Gao *et al*. reported that CD63, a membrane protein in MEVs, was able to bind to COP5, thereby achieving efficient capture of EVs^[111]^. Aldehyde-amine condensation can be used for EVs binding to aptamers, which can be used for precision medicine due to the extremely high affinity and specificity of aptamers^[112]^. Primary amines, sulfhydryl groups, and carboxyl groups in the membrane surface proteins of EVs offer possibilities for click chemistry. Different functional groups can be covalently linked for various purposes. According to reports, cerium oxide successfully binds to EVs via a thiol-maleimide reaction and modulates both innate and adaptive immunity in a collagen-induced arthritis model^[113]^.

### Physical engineering

The most commonly used strategies in physical engineering are membrane coating, membrane fusion, and loading drugs. Membrane coating can effectively increase the extensibility and functionality of EVs^[114]^. Chen *et al*. constructed a novel nanocarrier by fusing BEVs from attenuated *Salmonella* and MEVs from melanoma cells, and implanted poly (lactic-co-glycolic acid)-indocyanine green moiety into the above nanocarrier, which was capable of targeted delivery to tumor cells^[115]^.

Membrane fusion commonly refers to the merging of EVs and liposomes. Prior to this fusion, liposomal membranes are subject to functionalization, bestowing diverse abilities upon the EVs^[116]^. Their fusion can be achieved by several routes, including polyethylene glycol (PEG)-mediated fusion, co-incubation, and extrusion^[93,117,118]^. Hu *et al*. reported that a nanoparticle with bone-targeting ability and strong therapeutic effect was constructed by fusing bone-targeting effector-containing MEVs with bone therapeutic effector-containing liposomes via extrusion^[93]^. Piffoux *et al*. also found the fusion of PEG-triggered EVs with functionalized liposomes^[117]^. Lin *et al*. developed EV-liposome hybrids by simple incubation and successfully encapsulated a CRISPR-Cas9 expression vector^[118]^.

Drugs can be loaded into EVs in various ways, such as incorporation into nanomaterials for direct fusion with EVs, loading into nanomaterials for fusion with donor cells, or co-incubation with mother cells^[119-121]^. Drugs can also be loaded by electroporation, ultrasound, hypo-osmotic dialysis, and extrusion into EVs^[122-125]^. Jia *et al*. employed electroporation to encapsulate curcumin within EVs, successfully enabling its traversal of the blood-brain barrier and facilitating targeted drug delivery to the brain^[126]^. Lee *et al*. used lipid loading of various functional groups to achieve a wide variety of functionalities in EVs^[127]^. Saari *et al*. adeptly synthesized paclitaxel-loaded EVs via the incubation of EVs derived from Lymph Node Carcinoma of the Prostate (LNCaP) and PC-3 prostate cancer (PCa) cells with paclitaxel at a temperature of 22 °C for a period of 1 h^[119]^.

### AI-enabled construction of EVs

AI is an important component of computing. With the relentless progress of technology, it is enhancing numerous conventional industries and substantially boosting operational efficiency. Remarkably, there has been a proposal to leverage AI to empower the field of biomedicine^[128-130]^. Bai *et al*. introduced an innovative concept of applying AI to organoid research^[131]^. Incorporating AI holds the potential to significantly improve the design and construction of organoids, facilitating dynamic monitoring of culture conditions. We believe that similar approaches can accelerate research on EV engineering. Configuring distinct functional components to confer specific functionalities on EVs is a labor-intensive process. However, by applying predefined screening criteria, AI can rapidly identify the required components, thereby substantially enhancing efficiency. Furthermore, AI can offer more efficient modification techniques tailored to diverse cell sources and their respective characteristics^[132]^. Deep learning-based computational platforms also enable the precise prediction of bone-homing peptides and receptor-specific ligands, thereby enhancing the precision of targeted EV delivery^[133]^. In addition, the integration of multi-omics data through advanced AI algorithms allows for the systematic identification and selection of optimal therapeutic cargo, specifically tailored to address the complexities of bone-aging pathologies. Machine learning-driven optimization of EV production parameters improves scalability, yield, and batch-to-batch reproducibility, effectively addressing critical challenges in the manufacturing process. Furthermore, AI-powered real-time quality control systems that leverage high-throughput imaging and spectral analysis ensure stringent batch consistency and adherence to clinical standards. Collectively, these synergistic AI-driven strategies are poised to accelerate the development of safe, efficient, and clinically translatable engineered EVs, offering promising solutions for combating bone aging.

## BONE AGING MICROENVIRONMENT AND DISEASE

Bone is a dynamic organ whose structure and function are precisely regulated by various mechanisms, such as hormonal changes, oxidative stress, genetic factors, and chronic inflammation^[3,134]^. These processes become progressively altered with age, contributing to bone aging. The decline in estrogen levels disrupts the balance between osteoblasts and osteoclasts, resulting in increased bone resorption^[72]^. Oxidative stress, induced by the accumulation of reactive oxygen species (ROS), impairs osteoblast function and disrupts bone remodeling^[135]^. Genetic factors contribute to reduced bone mineral density and increased bone fragility, while chronic inflammatory microenvironments further promote bone resorption and suppress bone formation. The interplay of these factors contributes to a progressive deterioration in bone quality and heightened susceptibility to conditions such as OP and OA^[134]^. OP is characterized by reduced bone mineral density and microstructural deterioration, whereas OA is marked by articular cartilage degeneration and the formation of osteophytes. Therefore, a comprehensive understanding of bone aging and its underlying mechanisms is crucial for the development of innovative therapeutic strategies targeting OP and OA.

## APPLICATIONS OF EVs FOR OP

OP is a systemic bone metabolic disorder distinguished by reduced bone mass and disruption of skeletal microarchitecture. Scholars have identified that EVs play a pivotal role in the modulation of bone homeostasis and are currently recognized as promising targets for addressing OP^[136]^. Next, we summarize the roles of MEVs, BEVs, and PEVs in the OP [Table 1].

**Table 1:** EVs from various cell origins and their physiological significance in OP

**Type**	**Cell source of EVs**	**Function**	**Reference**
MEVs	DCs	Modulate local immune reactions	[137]
	ECs	Target bone tissue	[138]
	Osteoblasts	Promote differentiation of BMSCs into osteoblasts	[139]
	AFSCs	Attenuate steroid-induced oxidative stress	[140]
	DPSCs	Regulate telomerase activity and reduce osteoclast population	[141,142]
	USCs	Deliver osteoprotegerin to promote osteogenesis	[143]
	hiPSC-MSCs	Promote angiogenesis and bone regeneration	[144]
	BMSCs	Upregulate osteogenic effectors and decrease osteoclast numbers	[145]
	AMSCs	Inhibit activation of the NLRP3 inflammasome in osteoclasts	[146]
	UCMSCs	Enhance bone formation, diminish bone marrow fat accumulation, and mitigate bone resorption	[147]
BEVs	AKK	Promote bone formation	[67]
	PM	Inhibit osteoclast formation	[148]
PEVs	Yam	Promote osteoblast formation	[69]
	Pueraria lobata	Enhance BMSC differentiation via increased autophagy	[68]

EVs: Extracellular vesicles; OP: osteoporosis; MEVs: mammalian-derived extracellular vesicles; BEVs: bacteria-derived extracellular vesicles; PEVs: plant-derived extracellular vesicles; DCs: dendritic cells; ECs: endothelial cells; BMSCs: bone marrow mesenchymal stem cells; AFSCs: amniotic fluid stem cells; DPSCs: dental pulp stem cells; USCs: urine-derived stem cells; hiPSC-MSCs: human induced pluripotent stem cell-derived mesenchymal stem cells; AMSCs: amniotic mesenchymal stem cells; UCMSCs: umbilical cord mesenchymal stem cells; AKK: *Akkermansia muciniphila*; PM: *Pasteurella multocida*; NLRP3: NOD-, LRR-, and pyrin domain-containing protein 3.

### MEVs

**Figure 6:**
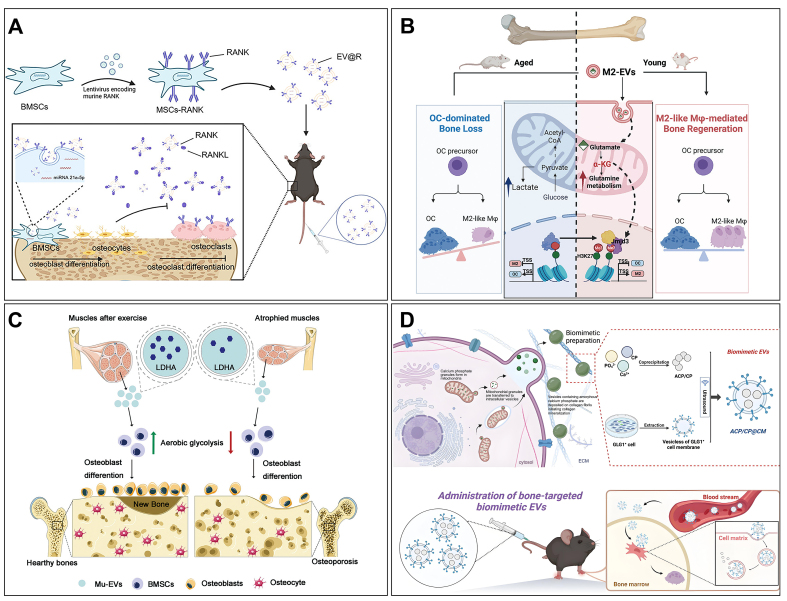
The role of MEVs in OP. (A) EVs expressing RANK from BMSCs were designed to remodel bone homeostasis for the treatment of OP. Copyright 2024 American Chemical Society; (B) EVs derived from M2 macrophages can promote the differentiation of osteoclast precursor cells into M2 macrophages and, through the upregulation of glutamate, reduce bone resorption and enhance bone repair. Copyright 2025 Elsevier Inc; (C) The condition of skeletal muscle directly affects the quantity and functionality of the EVs secreted by it. These EVs can be endocytosed by BMSCs, promoting the glycolysis process through the delivery of LDHA and subsequently regulating osteogenic differentiation. Copyright 2023 Elsevier Inc; (D) By leveraging biominerals and EVs with bone-targeting capabilities, a multifunctional biomimetic EV system can be engineered. This system not only enhances the osteogenic differentiation of BMSCs but also demonstrates the capacity to induce angiogenesis and promote collagen mineralization. Copyright 2025 American Chemical Society. MEVs: Muscle-derived extracellular vesicles; OP: osteoporosis; EVs: extracellular vesicles; RANK: receptor activator of nuclear factor kappa-B; BMSCs: bone marrow mesenchymal stem cells; LDHA: lactate dehydrogenase A.

Numerous studies have proved that MSCs of various origins can achieve therapeutic effects on OP by modulating bone homeostasis, stimulating vascular regeneration, and inhibiting the activation of nucleotide-binding domain-like receptor protein 3 (NLRP3) inflammatory vesicles^[149]^. Notably, Elashiry *et al*. observed that EVs secreted by DCs and carrying immunomodulators effectively inhibited osteoclast-mediated degenerative bone loss^[137]^. Song *et al*. noted that EVs secreted by vascular endothelial cells (ECs) possessed a heightened bone-targeting propensity in comparison to EVs derived from MSCs^[138]^. EVs derived from skeletal muscle, cartilage, or tendon better preserve the microenvironmental cues and functional signals of their source tissues. For instance, skeletal muscle-derived EVs are enriched in lactate dehydrogenase A (LDHA), which regulates glycolysis in BMSCs and promotes osteogenesis^[150]^. Compared to EVs derived from cell lines, this tissue specificity may offer superior therapeutic relevance for bone regeneration. In the following, we will detail the role of relevant MEVs in the OP treatment [Figure 6].

The expression of gene transcripts meticulously regulates the differentiation process of MSCs, directing them toward either adipocyte or osteoblast lineages^[151]^. However, MEVs from osteoblasts of OP patients showed that the reduced osteogenic rather than proliferative capacity of BMSCs, reflecting the influence of the bone microenvironment on cell metabolism in OP^[152]^. MEVs secreted by osteoblasts and osteoclasts first attracted the interest of researchers. Wei *et al*. reported that MEVs from osteoblasts can significantly enhance the osteogenic differentiation of BMSCs via positive feedback^[139]^. Li *et al*. also disclosed that osteoclast-secreted MEVs inhibited bone formation in osteoblasts by delivering miR-214-3p^[153]^. Therefore, strategies that inhibit MEV secretion by osteoclasts may offer a fresh perspective for the therapeutic intervention of OP. Similarly, EVs from different sources of stem cells have attracted the interest of researchers. For example, MEVs secreted by amniotic fluid stem cells (AFSCs) can treat OP by attenuating steroid-induced oxidative stress^[140]^. MEVs released by dental pulp stem cells (DPSCs) demonstrate the ability to regulate telomerase activity, consequently leading to a reduction in the count of tartrate-resistant acidic phosphatase (TRAP)-positive osteoclasts^[141,142]^. MEVs secreted by urine-derived stem cells (USCs) can deliver osteoprotegerin (OPG) and restore bone density for the OP treatment^[143]^. Other MEVs secreted by stem cells, including those from human induced pluripotent stem cells (hiPSC-MSCs)^[144]^, BMSCs^[145]^, adipose-derived mesenchymal stem cells (AMSCs)^[146]^, and umbilical cord mesenchymal stem cells (UCMSCs)^[147]^, have also shown efficacy in treating OP. Collectively, MEVs from diverse stem cell sources provide a completely new direction for OP therapy.

### BEVs

**Figure 7:**
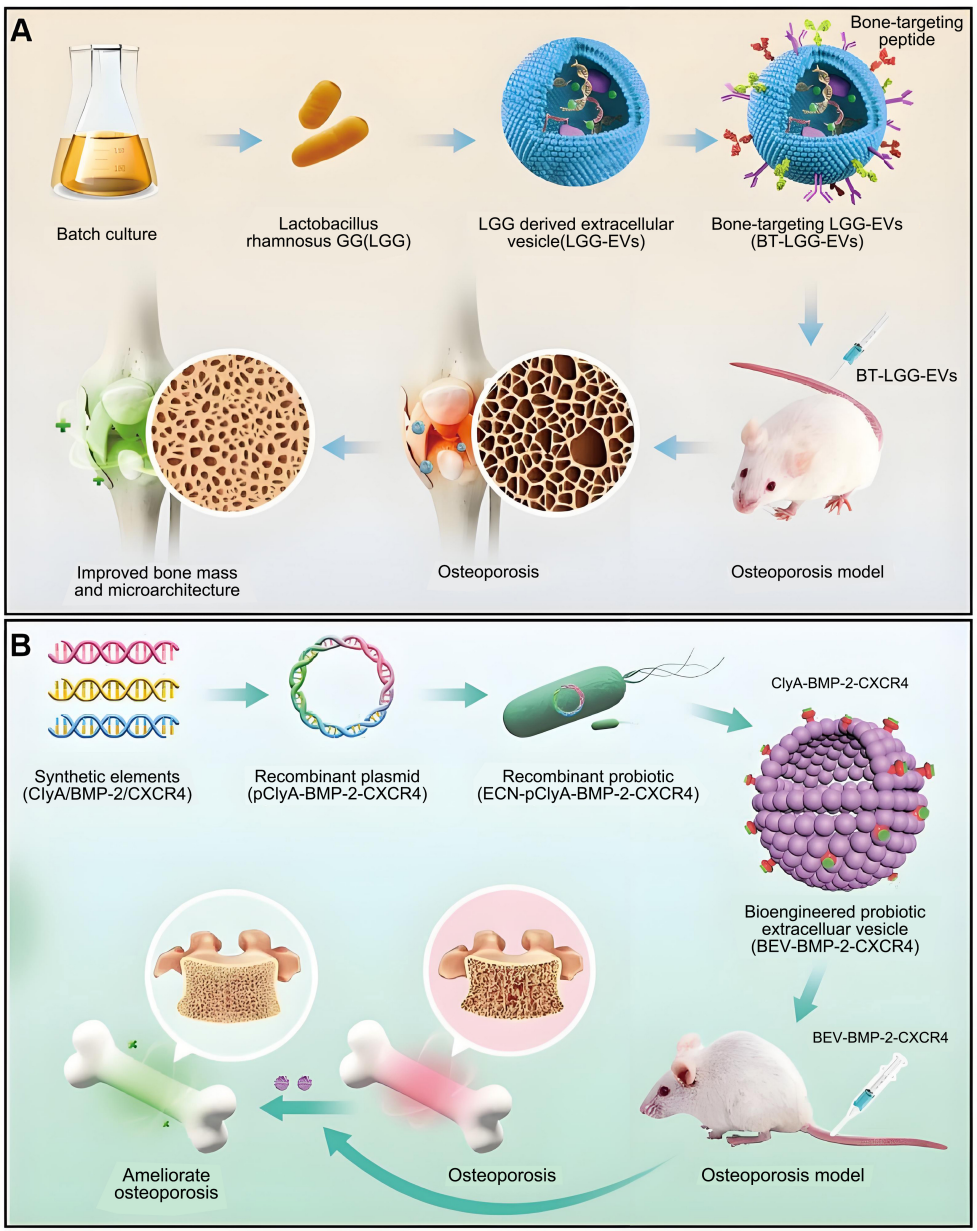
Overview of the role of BEVs in OP. (A) Anchor bone-targeting peptides onto the membrane of BEVs and achieve targeted treatment of osteoporosis by delivering endogenous miRNAs. Copyright 2023, Elsevier Ltd; (B) BMP-2 and CXCR4 components were synthesized to construct the recombinant plasmid pClyA-BMP-2-CXCR4, which facilitated the generation of the recombinant probiotic ECN-pClyA-BMP-2-CXCR4. This recombinant probiotic was then cultured to produce BEVs with bone-targeting and osteogenic capabilities. Copyright 2025, Wiley Periodicals LLC. BEVs: Bacterial extracellular vesicles; OP: osteoporosis; miRNAs: microRNAs; BMP-2: bone morphogenetic protein-2; CXCR4: C-X-C chemokine receptor type 4; pClyA: plasmid carrying cytolysin A; ECN: Escherichia coli Nissle 1917.

Fecal microbiota transfer is a developing therapeutic modality. Liu *et al*. demonstrated that transplanting gut microbes from children effectively treated ovariectomy (OVX)-induced OP in mice^[67]^. It was reported that AKK played an important role. Following this, the direct application of AKK to OVX-induced mice demonstrated therapeutic efficacy in addressing OP. Additionally, the therapeutic effect produced by AKK was significantly reduced after inhibiting the production of BEVs with GW4869. In contrast, direct administration of BEVs produced by AKK secretion to OVX-induced mice produced a therapeutic effect comparable to that of the parent strain. Therefore, it can be concluded that BEV produced by gut microorganisms can modulate bone homeostasis and thereby achieve a therapeutic effect on OP [Figure 7]. This finding reveals a novel intestinal-bone axis model of bone metabolism regulation.

Recently, Wang *et al*. also found that BEVs derived from the gut microorganism *Proteus mirabilis* (PM) could inhibit osteoclastogenesis and bone resorption^[148]^. Their study showed that PM-secreted BEVs induced mitochondrial dysfunction through collapse of mitochondrial membrane potential, elevated intracellular ROS levels, and modulation of Bax, Bcl-2, caspase-3, and cytochrome c expression, along with strong inhibition of miR-96-5p expression. Overall, PM-secreted BEVs possess osteoprotective functions, further enriching the emerging regulatory paradigm of BEVs-mediated gut-bone axis.

Each of the previously mentioned studies has illuminated a nascent paradigm in the coordination of bone metabolism, presenting a fresh therapeutic avenue for addressing OP. In particular, a solid foundation has been established for using probiotics, along with functional BEVs, in the OP treatment. We also expect that more BEVs will be discovered to further enrich the gut-bone axis regulation model.

### PEVs

**Figure 8:**
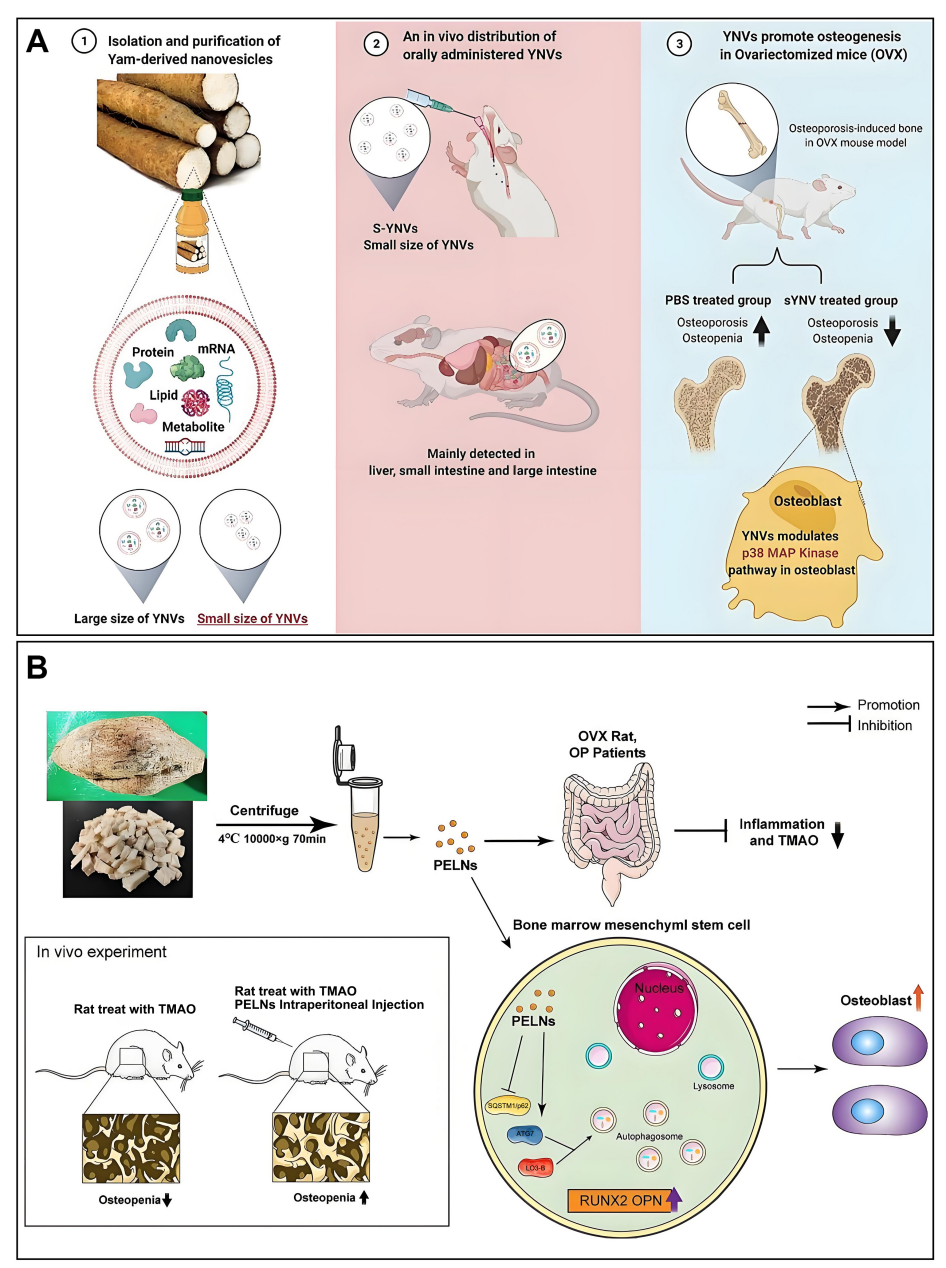
Overview of the role of PEVs in OP (A) EVs of yam origin, when administered orally, stimulate osteoblast formation, thereby effectively alleviating OP. Copyright 2023, Elsevier B.V.; (B) Pueraria lobata-derived EVs promote osteogenesis through enhanced autophagy. Copyright 2023, Elsevier B.V. PEVs: Plant-derived extracellular vesicles; OP: osteoporosis; EVs: extracellular vesicles; TMAO: trimethylamine-N-oxide; OVX: ovariectomy; PELNs: pueraria lobata-derived exosome-like nanovesicles; YNVs: yam-derived exosome-like nanovesicles; S-YNVs: small size of YNVs.

Previous studies have demonstrated the pivotal role of Chinese herbs in the treatment of OP^[154]^. Following the confirmation of the substantial role played by EVs in regulating the bone microenvironment between osteoblasts and osteoclasts, researchers shifted their focus to PEVs. Exosome-like vesicles derived from yam and Pueraria lobata have been reported to alleviate OP by modulating osteoblasts or by degrading trimethylamine-N-oxide (TMAO) and enhancing autophagy, as shown in Figure 8^[68,69]^.

In a recent breakthrough, Huang *et al*. achieved the successful isolation and characterization of PEVs from yam. The incorporation of these PEVs into the osteoblast culture medium yielded noteworthy results, notably stimulating the proliferation, differentiation, and mineralization of osteoblasts^[69]^. Subsequently, a comparative assessment of the osteogenic activity between PEVs and the saponin elements present in yam (specifically, components such as dioscin and diosgenin employed in OP treatment) unveiled the heightened osteogenic activity of PEVs. Notably, only a small amount of saponins could be extracted from a large amount of yam, whereas a large amount of PEVs could be extracted from a small amount of yam. Bioinformatics analysis further indicated that the osteogenic activity of PEVs is induced by p-p38/BMP-2-dependent activation of the Runx2 pathway. Finally, the OVX-induced OP symptoms were successfully alleviated by oral administration in OVX-induced OP mice. Surprisingly, the PEVs were able to maintain alkaline phosphatase (ALP) activity after storage at -80 °C for one year and remained non-biological toxicity at higher concentrations.

In 2023, Zhan *et al*. also found the ability of Pueraria lobata-derived EVs to alleviate OP. Furthermore, they identified TMAO, a metabolite associated with the gut microbiota, as a prospective target for OP therapy^[68]^. Similarly, the researchers successfully isolated and characterized the PEVs, which were added to the BMSC medium and successfully stimulated the differentiation and mineralization of BMSCs. Subsequently, researchers proceeded to analyze the differences in the fecal microbiota between normal individuals and those diagnosed with OP. The study revealed a significant increase in *Clostridium* in the feces of OP patients, accompanied by an anomalous elevation of TMAO in the serum of individuals afflicted with OP. Interestingly, *Clostridium* can produce TMAO, which inhibits osteoblast mineralization and the expression of associated osteogenic proteins. More interestingly, these effects were reversed after treatment with PEVs. Bioinformatics analysis suggested that the osteogenic activity of PEVs is mediated by regulating TMAO-regulated autophagy in BMSCs. Finally, the researchers again verified that PEVs could reverse TMAO-induced OP in a rat model.

## APPLICATIONS OF EVs FOR OA

**Figure 9:**
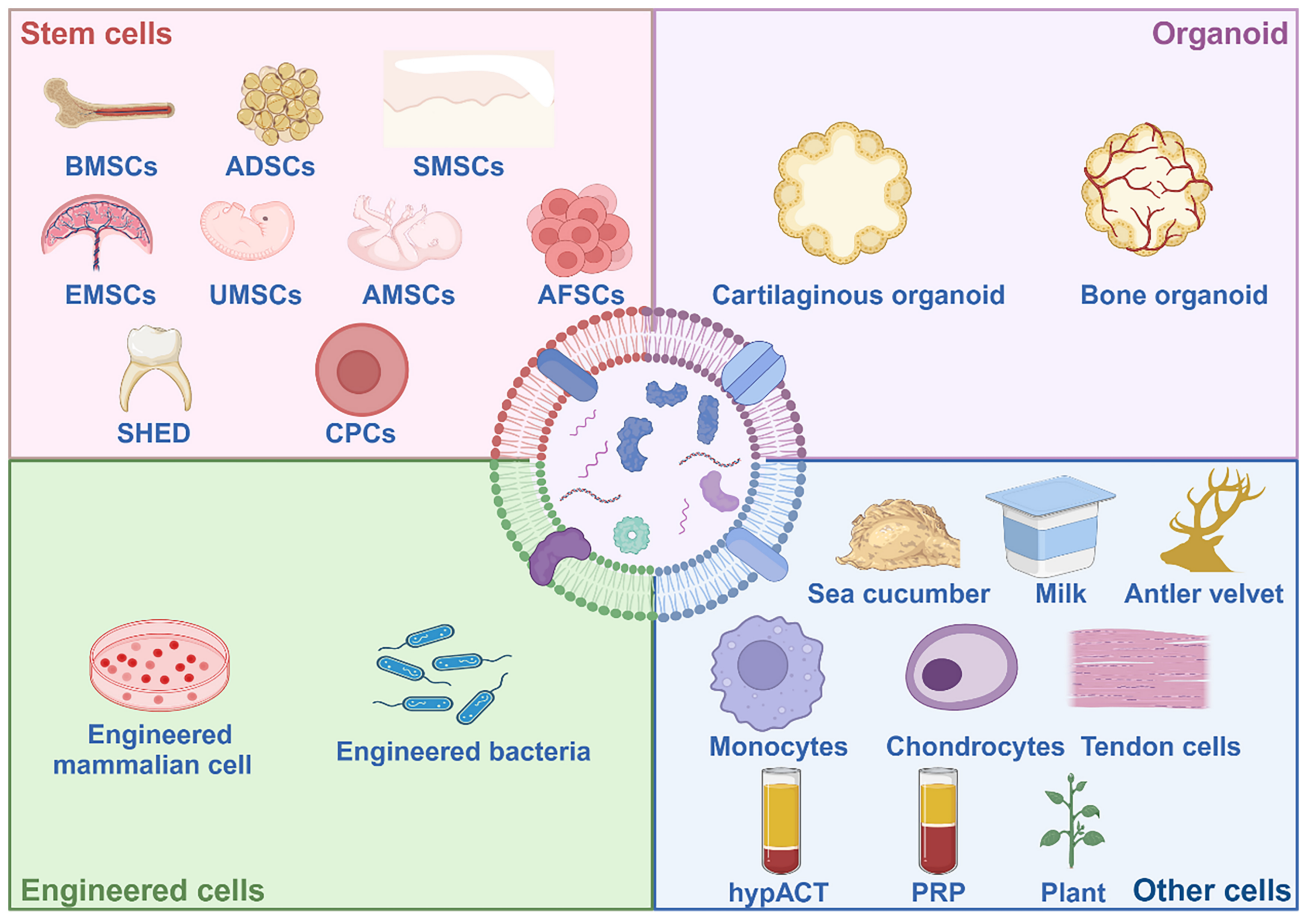
EVs from different sources for OA therapy. The existing and potential future donors of EVs are comprehensively introduced. EVs of organoid, bacterial, and plant may be potential therapeutic agents. Created in BioRender. Bigbone B. (2025) https://BioRender.com/wpbir74. EVs: Extracellular vesicles; OA: osteoarthritis; BMSCs: bone marrow mesenchymal stem cells; ADSCs: adipose-derived stem cells; SMSCs: synovium-derived mesenchymal stem cells; EMSCs: endometrium-derived mesenchymal stem cells; UMSCs: umbilical cord mesenchymal stem cells; AMSCs: amniotic mesenchymal stem cells; AFSCs: amniotic fluid stem cells; SHED: stem cells from human exfoliated deciduous teeth; CPCs: chondroprogenitor cells; PRP: platelet-rich plasma; hypACT: hyperacute serum.

EVs, as mediators of intercellular communication, have been extensively studied in the pathogenesis and diagnosis of OA^[155]^. Similarly, owing to their exceptional biological properties, EVs are regarded as promising biopharmaceuticals and drug delivery vehicles^[156]^. While MEVs from various origins exhibit considerable potential for the OA diagnosis and treatment, BEVs and PEVs have not yet garnered significant attention from researchers. This section focuses on the research progress of using MEVs in the treatment of OA. We also propose potential therapeutic strategies informed by previous studies, integrating the advantages of BEVs and PEVs [Figure 9].

### MEVs

Different sources of MEVs have shown varied roles in OA progression. MEVs have been extensively studied as biomarkers for OA diagnosis^[157]^. Here, we provide only a brief overview. EVs derived from blood, synovial fluid within the joint cavity, and tissue sources in patients with OA exhibit distinct differences compared to those from healthy individuals^[158-160]^. These differences offer significant potential for early OA diagnosis, especially in cases where symptoms are not yet apparent. Notably, EVs derived from synovial fluid are rich in substances secreted by joint structures, reflecting the status of OA. They also have the potential to identify different OA subtypes based on their distinct contents^[161]^. It is important to note that EV-based diagnostic methods primarily target specific joints, and their reliability diminishes when multiple joints are involved. This section focuses on summarizing the roles of different MEVs in OA treatment [Table 2].

**Table 2:** The role of MEVs in the treatment of OA

**Cell source of EVs**	**Functions**	**Reference**
BMSCs	Regulate macrophage polarization, inhibit inflammation in OA, and suppress IL-1β-induced chondrocyte senescence and apoptosis	[162-164]
ADSCs	Inhibit M1 macrophage infiltration in the synovium and suppress the inflammatory response by targeting the NF-κB signaling pathway	[165,166]
SMSCs	Inhibit PTEN expression to suppress inflammation and apoptosis; target HMGB1 to alleviate the progression of OA	[167,168]
SHED	Inhibit chondrocyte inflammation	[169]
AMSCS	Promote macrophage polarization and inhibit inflammatory T cells	[170]
UMSCs	Inhibit chondrocyte ROS production and regulate macrophage polarization	[171,172]
EMSCs	Increase type II collagen synthesis and inhibit ADAMTS5 expression to protect chondrocytes and suppress IL-1β-induced MMP13 production	[173,174]
AFSCs	Promote cartilage repair through TGF-β	[175]
hypACT	Promote COL2A1 and ACAN expression and enhance chondrocyte anabolic activity	[176]
PRP	Inhibit TNF-α and activate the Wnt/β-catenin signaling pathway	[177]
Monocytes	Enhance chondrocyte differentiation and function	[178]
Chondrocytes	Inhibit inflammation and regulate mitochondrial function	[179]
Tendon cells	Promote MSC differentiation into tendon cells	[180]
Sea cucumber	Inhibit synovial inflammation	[181]
Antler velvet	Promote MSC proliferation and inhibit inflammation and MSC senescence	[182]
Milk	Regulate immunity and inhibit inflammation	[183]

MEVs: Mesenchymal extracellular vesicles; OA: osteoarthritis; BMSCs: bone marrow mesenchymal stem cells; ADSCs: adipose-derived stem cells; SMSCs: synovium-derived mesenchymal stem cells; SHED: stem cells from human exfoliated deciduous teeth; AMSCs: amniotic mesenchymal stem cells; UMSCs: umbilical cord mesenchymal stem cells; EMSCs: endometrium-derived mesenchymal stem cells; AFSCs: amniotic fluid stem cells; hypACT: hyperacute serum; PRP: platelet-rich plasma; IL-1β: interleukin-1 beta; NF-κB: nuclear factor kappa-light-chain-enhancer of activated B cells; PTEN: phosphatase and tensin homolog; HMGB1: high mobility group box 1; ROS: reactive oxygen species; ADAMTS5: a disintegrin and metalloproteinase with thrombospondin motifs 5; MMP13: matrix metalloproteinase-13; TGF-β: transforming growth factor beta; COL2A1: collagen type II alpha 1 chain; ACAN: aggrecan; TNF-α: tumor necrosis factor alpha; MSCs: mesenchymal stem cells.

Stem cell-based treatments for OA have proven to be highly promising approaches^[184]^. However, tumorigenicity and immune rejection continue to be significant obstacles in stem cell therapy^[185,186]^. The advent of EVs presents an optimal solution to these issues. He *et al*. demonstrated that EVs derived from BMSCs can upregulate COL2A1 expression and downregulate matrix metalloproteinase-13 (MMP13) expression, thereby promoting chondrocyte repair and maintaining extracellular matrix stability^[187]^. Meanwhile, Zhang *et al*. found that BMSC-derived EVs could attenuate the progression of OA by modulating macrophage polarization^[164]^. Woo *et al*. demonstrated that EVs derived from adipose mesenchymal stem cells (ADSCs) effectively inhibit M1 macrophage infiltration in the synovium, thereby reducing chondrocyte catabolism^[165]^. It is worth mentioning that ADSC-EVs have demonstrated an extremely strong therapeutic role in early OA. Researchers have also found that EVs derived from synovial mesenchymal stem cells (SMSCs) can deliver miR-129-5p and alleviate OA progression by targeting high-mobility group box 1^[167]^. Luo *et al*. found that EVs of human exfoliated deciduous teeth stem cells (SHED) also delivered MiR-100-5p to inhibit inflammation in OA^[169]^. EVs derived from amniotic membrane mesenchymal stem cells (AM-MSCs), UMSCs, embryonic mesenchymal stem cells (EMSCs), and AFSCs have also shown therapeutic potential for OA^[170,171,173,175]^.

Additionally, EVs from body fluids, adult cells, marine organisms, and antler sources have attracted the attention of researchers. These donor cells tend to have simpler functions than stem cells, which also means their quality is more controllable. Blood-derived EVs have been shown to reduce pain and inflammation by intra-articular injection^[188]^. Macrophages are closely associated with the progression of OA. Bai *et al*. found that EVs derived from M2-type macrophages, which deliver SOX9 to host cells, enhance chondrocyte function^[178]^. Cartilage is a crucial component of the joint, responsible for maintaining extracellular matrix homeostasis. The state of chondrocytes significantly influences the progression of OA. Zheng *et al*. demonstrated that EVs originating from cartilage can alleviate OA by modulating mitochondrial and immune responses^[179]^. Similarly, the tendon is a structure that maintains joint stability, and its disruption often leads to OA^[189]^. Xu *et al*. demonstrated that EVs derived from tendon cells can promote the differentiation of mesenchymal stem cells into tendon cells via transforming growth factor β (TGF-β) signaling^[180]^. Marine organisms are rich sources of biomaterials, with sea cucumbers particularly attracting researchers’ interest due to their strong regenerative abilities. Jo *et al*. employed collagenase treatment on lyophilized sea cucumber extracellular matrix to obtain EVs and demonstrated their ability to decrease COX-2 expression and reduce synovial inflammation^[181]^. This study demonstrates the potential of marine-derived EVs in treating OA. Antler velvet, which is a unique organ capable of periodic complete regeneration, has also been studied. Lei *et al*. discovered that EVs derived from deer antlers can alleviate OA by reducing P16 and P21 protein levels, β-galactosidase activity, and senescence-associated secretory phenotype gene expression^[182]^.

With the rapid advancement of organoid technology, EVs derived from organoids have garnered increasing attention. These EVs, obtained through 3D culture, are regarded as more representative of *in vivo* metabolism^[190]^. Recently, EVs derived from retinal organoids have been shown to protect retinal epithelial cells from damage by regulating fatty acid metabolism^[191]^. A comparison between organoid-derived EVs (OEVs) and human embryonic stem cell-derived EVs revealed that OEVs exhibit greater selectivity in protein loading. It is worth noting that OEVs are enriched with proteins involved in immune regulation and retinal development. This study demonstrated that OEV has better efficacy than 2D culture-derived EVs. Researchers have successfully constructed cartilage-like organs and achieved cartilage repair by utilizing induced pluripotent stem cells (iPSCs) and BMSCs^[192,193]^. However, cartilage-like organ-derived EVs have not been thoroughly investigated. These OEVs may offer enhanced therapeutic effects and provide additional insights into the pathogenesis of OA. Further exploration by researchers is warranted.

**Figure 10:**
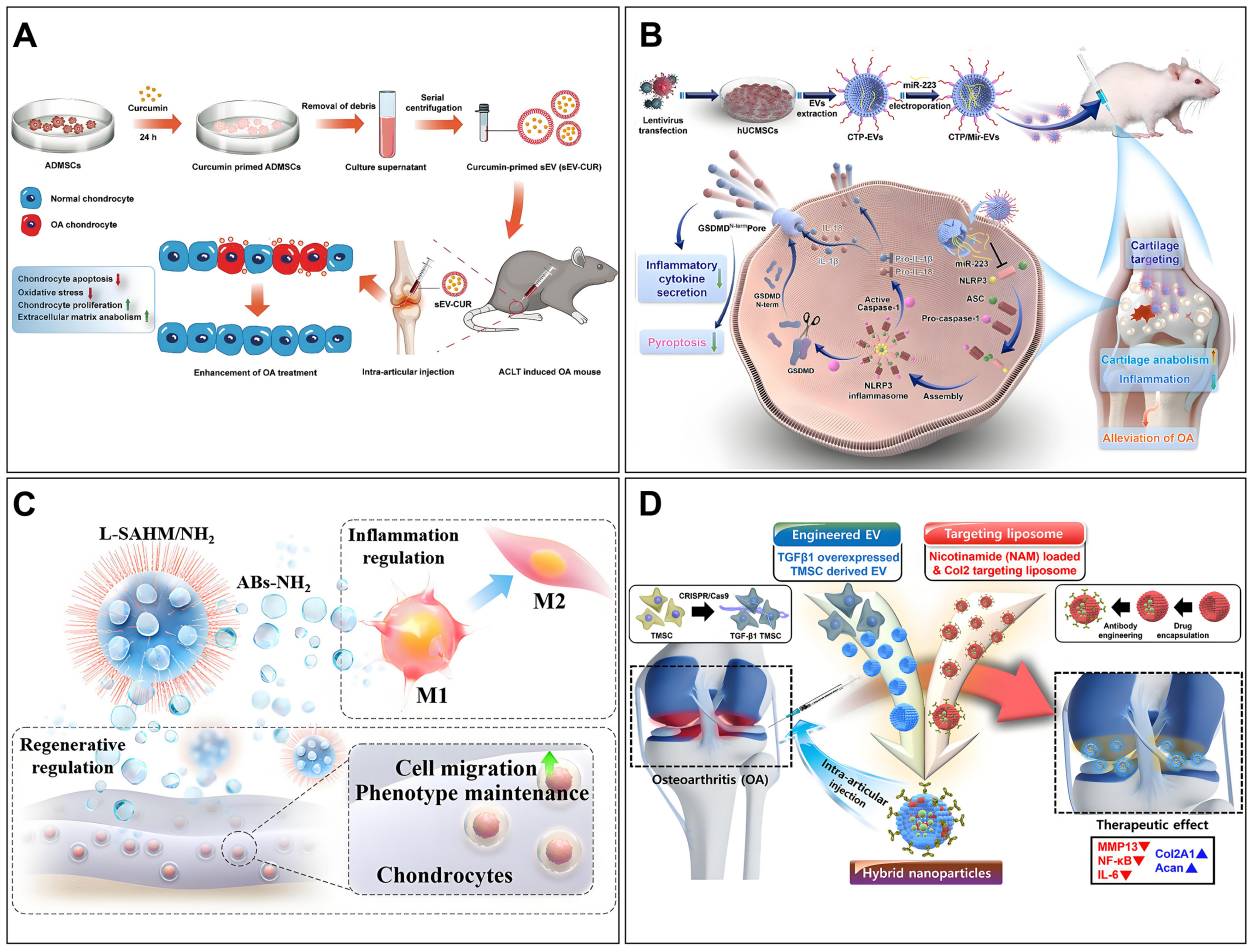
MEVs for OA therapy. (A) MEVs loaded with curcumin are obtained through co-culture with ADSCs, and these MEVs exhibit excellent cartilage-protective effects. Copyright 2022 Springer Nature; (B) Exogenous miR-223 is loaded into MEVs via electroporation to enhance their therapeutic efficacy, followed by surface modification with a type II collagen-targeting peptide to confer targeting capability. These bifunctional MEVs exhibit strong therapeutic effects against OA. Copyright 2023 Elsevier; (C) An injectable multifunctional therapeutic system was developed by encapsulating T cell-derived EVs into lubricative hydrogel microspheres. The inflammatory microenvironment was effectively modulated, cartilage homeostasis was promoted, and cartilage surface friction was reduced by this system, thereby advancing cartilage remodeling and OA treatment. Copyright 2024 American Chemical Society; (D) The hybrid nanoparticles, fabricated through the hybridization of nicotinamide-encapsulated and Col2A1 antibody-modified liposomes with TGF-β1-overexpressing EVs, were demonstrated to exhibit targeted delivery, anti-inflammatory, and cartilage-protective capabilities, effectively mitigating the progression of OA. Copyright 2024 American Chemical Society. MEVs: Macrophage-derived extracellular vesicles; OA: osteoarthritis; ADSCs: adipose-derived stem cells; miR: microRNA; EVs: extracellular vesicles; Col2A1: type II collagen alpha 1 chain; TGF-β1: transforming growth factor beta 1.

While natural MEVs have shown promising therapeutic potential, modified EVs offer superior targeting, delivery efficiency, and therapeutic efficacy. The modification methods have been outlined previously. Here, we focus on the application of engineered EVs in OA. Current strategies for treating OA using engineered MEVs are broadly categorized into targeting enhancement and therapeutic capacity improvement [Figure 10]. Xu *et al*. engineered E7-MEVs by fusing the MSC-binding peptide E7 to the membrane protein LAMP2B of MEVs, thereby conferring targeting ability^[194]^. E7-MEVs successfully delivered kartogenin to synovial MSCs, promoting chondrocyte formation. Additionally, chondrocyte-affinity peptide (CAP) was fused with membrane proteins to create cartilage-targeted CAP-MEVs^[104]^. CAP-MEVs specifically deliver miR-140 to chondrocytes, thereby effectively inhibiting cartilage-degrading proteases. Additionally, enhancing the anti-inflammatory capacity of MEVs is also a powerful therapeutic strategy. A large number of herbs contain anti-inflammatory activity, such as curcumin, icariin, and quercetin. However, stability and bioavailability issues often limit the application of these compounds. Numerous studies have demonstrated that EVs obtained from BMSCs and ADSCs pretreated with curcumin exhibit enhanced cartilage protection^[195,196]^. Similarly, EVs derived from MSCs pretreated with hypoxia exhibit high expression of specific miRNAs, thereby enhancing cartilage repair^[197,198]^. It is evident that the delivery of specific miRNAs or therapeutic agents can significantly enhance therapeutic efficacy. Researchers have utilized gene transfection and electroporation to deliver miR-126-3p, miR-127-3p, miR-361-5p, and kartogenin to mitigate the progression of OA^[194,199-201]^.

### BEVs

**Figure 11:**
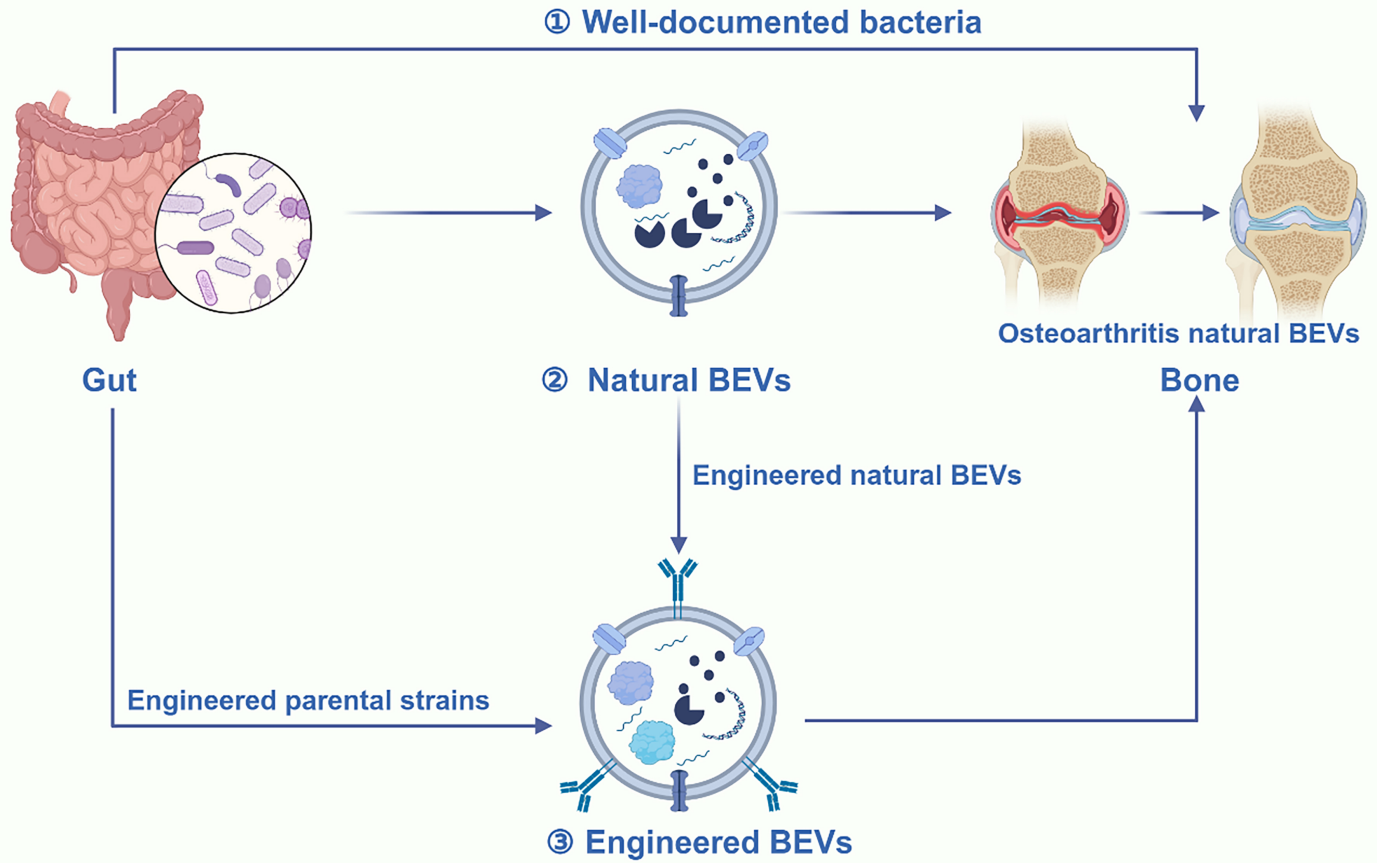
Both natural and engineered BEVs are promising therapeutic agents for OA therapy. BEVs play a crucial role in the gut-bone axis communication. Therefore, besides using these bacteria directly to alleviate OA, utilizing EVs derived from these bacteria may offer a superior treatment option. Additionally, both engineered strains and BEVs can be used to produce functional BEVs. These engineered BEVs exhibit enhanced properties, including reduced toxicity, high yield, targeted delivery to chondrocytes, and improved cartilage repair capabilities, thus providing greater potential for OA treatment. Created in BioRender. Bigbone B. (2025) https://BioRender.com/cw92itq. BEVs: Bacterial extracellular vesicles; OA: osteoarthritis; EVs: extracellular vesicles.

In recent years, BEVs have seen increasing application in bone diseases^[202]^. Using synthetic biology techniques, Liu *et al*. successfully displayed BMP-2 and CXCR4 on the membrane surface of BEVs, endowing them with both targeting and therapeutic capabilities^[203]^. This study indicates that BEVs hold great promise for bone disease treatment due to their highly modifiable properties. Additionally, the ease of culturing bacteria at an industrial scale enhances their potential for future clinical applications. Notably, both natural and engineered BEVs have shown therapeutic potential for OP^[72]^. However, there is a notable lack of studies on the use of BEVs for OA treatment. In this section, we propose potential strategies for utilizing BEVs in OA treatment to advance the field. Rapid advancements in synthetic biology have enabled effective customization of BEVs^[86]^. For example, we engineered BEVs by fusing CAP to their membrane proteins, thereby enhancing targeting ability. This approach facilitates the precise delivery of a large number of carriers to chondrocytes. Electroporation is employed to load drugs or miRNAs into BEVs for therapeutic effects. This method represents a promising strategy for OA treatment using BEVs. Additionally, strategies targeting macrophage polarization and immune response modulation have been shown to effectively alleviate OA^[171]^. However, the use of EVs derived from human umbilical cord mesenchymal stem cells (hUCMSCs) still faces the common issue associated with MEVs, such as low yield. It is clear that the emergence of BEVs perfectly addresses this issue. We can easily polarize macrophages by displaying IL-4 on the membrane surface of BEVs^[204]^. Simultaneously, by displaying CAP on the membrane surface of BEVs, we can engineer them with the ability to target cartilage for immune modulation. Furthermore, different regulators can be presented on BEV membranes to modulate mechanisms involved in OA pathogenesis, enhancing therapeutic effects. Additionally, Liu *et al*. demonstrated that BEVs from the gut of children exhibit a natural therapeutic role in OP treatment^[67]^. This study proposes a novel mechanism of gut-bone communication, laying the foundation for the development of oral BEVs. The role of gut microbiota in influencing OA progression through the gut-bone axis is well established^[205,206]^. Similarly, it is valuable for researchers to investigate whether BEVs derived from gut microbes have therapeutic potential for OA [Figure 11].

### PEVs

Currently, strategies for treating OA based on PEVs are virtually non-existent. However, it has been confirmed that MEVs with plant pretreatment possess enhanced cartilage repair capabilities^[195,196]^. Anti-inflammatory treatment is a crucial component of OA management. Recent research indicates that PEVs exhibit strong anti-inflammatory capabilities [Table 3].

**Table 3:** Mechanisms of action of anti-inflammatory PEVs

**Source of EVs**	**Mechanism**	**Reference**
Ginger	Promote IL-10 and inhibit TNF-α, IL-6, and NLRP3	[207]
Grapefruit	Regulate macrophage HO-1 expression and inhibit IL-1β and TNF-α	[208]
Cabbage	Inhibit macrophage COX-2, IL-1β, and IL-6 expression	[209]
Blueberry	Reverse the effects of TNF-α and IL-6	[210]
Grape Nut Garlic Ginseng	Inhibit TNF-α and NF-κB Inhibit the TNF-α pathway and reduce TNFPQ protein expression Inhibit NLRP3 inflammasome activation Promote M1 macrophage polarization to M2 macrophages	[211] [212] [213] [138]

PEVs: Plant-derived extracellular vesicles; IL: interleukin; IL-1β: interleukin-1 beta; IL-6: interleukin-6; IL-10: interleukin-10; TNF-α: tumor necrosis factor-alpha; NLRP3: NOD-like receptor family pyrin domain containing 3; HO-1: heme oxygenase-1; COX-2: cyclooxygenase-2; NF-κB: nuclear factor kappa-light-chain-enhancer of activated B cells; M1/M2 macrophages: classically activated macrophages / alternatively activated macrophages.

Notably, EVs derived from ginger have demonstrated therapeutic effects in conditions such as inflammatory bowel disease, autoimmune liver disease, and rheumatoid arthritis^[61,207,214]^. It is anticipated that ginger-derived EVs may help mitigate the progression of OA. Given the abundance of plant sources, many underexplored plants, such as quercetin, warrant further investigation. The fusion of PEVs and BEVs presents a highly promising direction. PEVs possess strong anti-inflammatory capabilities, while BEVs can be easily endowed with targeting abilities. The hybridized EVs formed through this fusion combine both anti-inflammatory and targeting properties, effectively avoiding the inefficiency associated with cargo loading via electroporation mentioned in the previous section. However, research on PEVs-based treatments for OA has been relatively slow and requires more extensive exploration by researchers.

## ADVANTAGES AND CHALLENGES OF EVs

**Figure 12:**
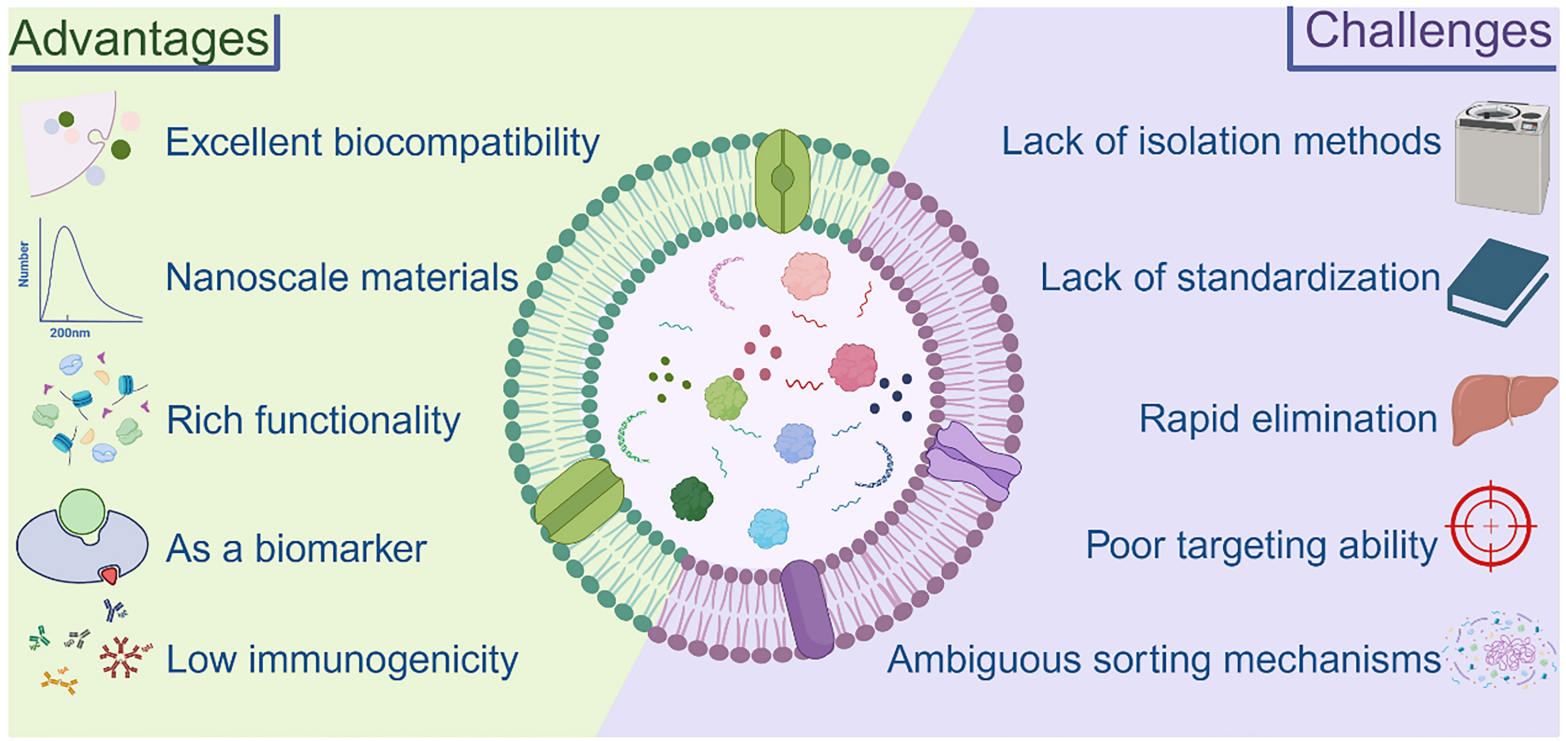
The main strengths of EVs as they exist today and the challenges they will face. Created in BioRender. Bigbone B. (2025) https://BioRender.com/gyl8jqc. EVs: Extracellular vesicles.

EVs exhibit distinct characteristics depending on their species of origin, yet they undoubtedly hold great potential in biomedicine. MEVs have been studied for a considerable time, while recent attention has shifted toward BEVs and PEVs. Here, we briefly outline the benefits and challenges of EVs to support the field’s rapid growth [Figure 12].

### Advantages

Due to their structural characteristics, the three different classes of EVs share several common advantages. Their nanoscale size and good biocompatibility make them a new generation of drug delivery carriers and hold great promise for vaccine delivery^[215,216]^. Additionally, EVs are easily modifiable, with many studies demonstrating enhanced therapeutic effects through various modification strategies^[93]^. Meanwhile, EVs exhibit diverse functional roles and have been shown to participate in multiple disease contexts^[217]^.

This makes BEVs an exceedingly appealing choice for prospective industrial production, holding considerable promise for commercialization. Compared with MEVs, BEVs can directly modify parental strains without worrying about ethical issues. There is no doubt that BEVs can be used as biomarkers for bacterial infections. This provides a new direction for rapid clinical diagnosis.

The most significant features of PEVs are that they are environmentally friendly, non-polluting, and sustainably available with few ethical concerns. Meanwhile, large-scale production of PEVs is relatively easy due to their wide range of sources and easy scale production of plants^[218]^. Investigations have consistently demonstrated that the effectiveness of PEVs remains unaltered post oral administration. Additionally, these PEVs showcase exceptional stability when stored for extended periods at -80 °C, establishing an exceedingly favorable scenario for potential clinical applications and for the commercial transport^[69]^. It is worth mentioning that for Chinese herbal medicines, there are long-term medication experiences and corresponding efficacy references, and the combination of EVs can effectively guide researchers to discover new disease targets^[68]^.

### Challenges

A common limitation of EVs is the lack of efficient standard isolation and purification methods, which may further affect the reproducibility of experiments. Additionally, EVs exhibit limited targeting ability and low efficacy; however, engineering modifications can be a more effective solution to these problems^[219]^. Meanwhile, the complexity of the contents encapsulated in EVs and the variability of EV contents from different sources lead to safety uncertainties, which must be overcome for successful clinical translation. It is worth mentioning that the mechanism of EV content sorting remains unclear and requires further investigation and validation^[149]^. Although EVs are known to carry miRNAs and other bioactive molecules, recent studies have raised concerns about the actual copy number per vesicle. It has been estimated that the average number of specific miRNAs per EV is often less than one, which brings into question their physiological relevance under normal dosing^[220]^. However, evidence also suggests that sustained exposure and paracrine amplification may overcome this limitation^[221]^. Moreover, EV uptake efficiency varies by cell type and tissue, with phagocytic cells showing higher endocytosis rates, while non-phagocytic cells exhibit more limited internalization. These factors highlight the need for dose optimization and enhanced delivery systems in EV-based therapies.

For BEVs, the lack of specific proteins presents a considerable challenge in characterization^[41]^. The cultivation of bacteria, as outlined in the protocol for engineering modifications of parental strains and the subsequent overexpression of heterologous proteins, demands low-temperature induction. Nevertheless, this requirement proves counterproductive for attaining industrial-scale production and impeding the commercialization efforts of BEVs^[85]^. In addition, the pathogenicity factors contained in BEVs are important issues that need to be addressed.

The most significant disadvantage of PEVs is the long growth cycle of the plant, which is also not conducive to future industrialization and commercialization. The lack of appropriate biomarkers for PEVs also poses a challenge to their characterization, hindering their development to some extent^[58]^. Meanwhile, there are still few studies on the storage conditions of PEVs, and their efficacy may be affected by various aspects such as temperature and pH.

## CONCLUSIONS AND PROSPECTS

This review provides a comprehensive summary of the latest advancements in MEVs, BEVs, and PEVs within the context of bone aging research. Although MEVs significantly influence the progression of bone metabolism, their yield and purity remain relatively low, limiting their further development^[149,155,222]^. Consequently, more readily accessible BEVs and PEVs have attracted our attention. Recent studies have substantiated that BEVs and PEVs can effectively modulate bone metabolism^[67,68]^. Furthermore, due to their higher yield and more closely aligned *in vivo* metabolism compared to traditional MEVs, OEVs may develop into a crucial component of MEVs^[223]^. Accordingly, we propose the innovative concept of utilizing OEVs to treat bone aging-related diseases, while also summarizing the therapeutic advantages of each EV type.

Although various types of EVs possess distinct characteristics and advantages, their selection for therapeutic purposes should primarily be guided by biological functions, disease context, and the immunological profile of the recipient tissue. Optimal administration strategies should be tailored to disease-specific requirements. Local injection ensures high drug concentrations at the target site with minimal systemic clearance, offering clear benefits for joint-focused disorders such as OA. Conversely, systemic infusion provides extensive biodistribution, which may be preferable for mitigating generalized bone loss in OP. Beyond serving as anti-bone-aging nanocarriers, EVs can be combined with functional biomaterials - including mesoporous inorganics, metallic scaffolds, and hydrogels - to enable controlled release and synergistic therapeutic effects, thereby expediting tissue regeneration.

Moreover, MEVs can carry a range of bioactive molecules from parent cells, effectively reflecting their cellular state^[224]^. By comparing and analyzing the content differences in MEVs under healthy and aging conditions, and monitoring their changes, the stage of bone aging diseases can be accurately assessed^[225,226]^. The concentration of specific biomarkers in MEVs is significantly correlated with disease stage, aiding in the determination of the patient’s pathological state and the timely adjustment of treatment strategies for precision medicine^[226]^. Thus, MEVs show great potential as biomarkers for diagnosing bone aging diseases.

Bacteria benefit from established high-density culture methods and advanced gene editing technologies, facilitating the large-scale isolation and purification of BEVs^[136,222]^. Furthermore, the rapid advancement of synthetic biology offers vast potential for the customization of engineered BEVs in bone aging^[227]^. Furthermore, we present the concept of AI-enabled engineering modifications of EVs, which may represent a promising direction for EV engineering. Indeed, BEVs possess the capacity to cross the intestinal epithelial barrier and interact with immune cells to initiate immune regulation^[228-230]^. Consequently, employing BEV-based oral bacterial strategies to modulate immune cells may represent a promising avenue for OP treatment. One of the best-characterized EV-mediated mechanisms in bone remodeling involves receptor activator of nuclear factor kappa B-ligand (RANKL)- and receptor activator of nuclear factor kappa B (RANK)-containing EVs. Recent studies have demonstrated that EVs can transfer RANKL to osteoclast precursors, promoting osteoclastogenesis via reverse signaling^[231]^. This EV-mediated pathway plays a central role in bone resorption, particularly in OP, and may serve as a target for therapeutic intervention using decoy receptors or neutralizing strategies.

PEVs, readily sourced and sustainably obtained, facilitate the efficient large-scale isolation and purification process^[58,218]^. They offer exceptionally high safety profiles and have been utilized as biotherapeutics or drug delivery vehicles^[61,232]^. Furthermore, compared to natural products, PEVs demonstrate enhanced stability, wider applicability, and reduced side effects. Relative to BEVs, PEVs offer advantages in isolation and purification, immunogenicity, biocompatibility, and source availability^[72]^. Their potent anti-inflammatory effects have been substantiated in the context of autoimmune diseases. Therefore, whether utilized as drug delivery vehicles or biotherapeutics, PEVs present significant potential for bone aging.

As organoid research advances rapidly, OEVs are poised to receive increased attention. Trentesaux *et al*. have employed synthetic biology to engineer organoids and augment their regenerative capabilities^[233]^. Our team has likewise introduced the concept of AI-enabled organoids^[131]^. By leveraging AI to guide the development of organoids and integrating it with synthetic biology techniques, we can customize OEVs for bone aging.

Recent studies have revealed that EVs can regulate the homeostasis of key organelles in recipient cells, thereby offering novel therapeutic pathways beyond traditional miRNA/protein signaling. Mitochondrial-derived EVs can transfer mitochondrial components - or even intact mitochondria - to recipient osteoblasts and BMSCs, restoring their bioenergetic function and alleviating oxidative stress^[234]^. Additionally, cells experiencing lysosomal dysfunction secrete large EVs containing mitochondrial fragments, which are taken up by macrophages, altering their metabolic and immune profiles^[235]^. EVs also modulate autophagy and mitophagy through PI3K/Akt/mTOR-regulated miRNAs, maintaining organelle quality control. In osteogenic models, MSC-derived EVs enhance autophagy, promoting the survival and differentiation of osteoblasts under oxidative stress. Furthermore, EVs can influence lysosomal and endoplasmic reticulum stress responses, suppressing the activation of the NLRP3 inflammasome in inflammatory microenvironments^[236]^. Finally, EVs derived from muscle or stem cells can deliver metabolic enzymes to reprogram the energy metabolism of BMSCs, supporting bone regeneration processes^[150]^.

Despite the promising therapeutic potential of EVs in bone aging-related disorders, several critical challenges remain unresolved. Currently, standardized protocols for the isolation and purification of diverse EV subtypes are lacking, and multiple variables within the preparation process can significantly influence their bioactivity and stability. These limitations impede both fundamental research and clinical translation. Consequently, it is imperative to establish robust and standardized methods for EV separation and purification, systematically elucidate their underlying biological mechanisms, and integrate well-designed clinical trials to facilitate the advancement of EV-based therapies for bone aging diseases.

## DECLARATIONS

### Copyright

© The Author(s) 2025.
